# Unveiling Dipolar Interaction‐Driven Magnetic Field Inhomogeneities in T_2_ MRI Contrast Agents

**DOI:** 10.1002/advs.202510356

**Published:** 2025-11-04

**Authors:** Pelayo García‐Acevedo, Yolanda Piñeiro, Juan Gallo, Pedro Ramos‐Cabrer, Ramón Iglesias‐Rey, José Rivas, Manuel Bañobre‐López

**Affiliations:** ^1^ NANOMAG Laboratory, Applied Physics Department, iMATUS Materials Institute and Health Research Institute of Santiago de Compostela (IDIS) Universidade de Santiago de Compostela Santiago de Compostela 15782 Spain; ^2^ Neuroimaging and Biotechnology Laboratory (NOBEL), Clinical Neurosciences Research Laboratory (LINC) Health Research Institute of Santiago de Compostela (IDIS) Santiago de Compostela 15706 Spain; ^3^ Advanced (magnetic) Theranostic Nanostructures Lab International Iberian Nanotechnology Laboratory Braga 4715‐330 Portugal; ^4^ Center for Cooperative Research in Biomaterials (CIC biomaGUNE) Basque Research and Technology Alliance (BRTA) Donostia‐San Sebastián 20014 Spain

**Keywords:** contrast agents, dipolar interactions, magnetic nanoparticles, magnetic resonance imaging, ultra‐high field‐MRI

## Abstract

Modulating local magnetic field inhomogeneities, combined with ultra‐high‐field MRI (UHF‐MRI) is a promising strategy to enhance the performance of next‐generation T_2_ contrast agents (CAs). Although dipolar interactions likely contribute to contrast enhancement, their role in magnetic inhomogeneities and proton dephasing remains poorly understood, limiting further optimization. In this study, the fundamental role of dipolar interactions on the modulation of the transverse relaxivity (r_2_) of iron oxide‐based CAs is demonstrated by shaping local magnetic field inhomogeneities. A nanoscale‐distance‐tuned model system is developed by coating superparamagnetic iron oxide nanoprobes with silica shells of increasing thickness, thereby modulating the intensity of the dipolar interactions. An exponential dependence of r_2_ on dipolar interaction strength is observed, with a sharp initial increase followed by a plateau as interactions reached their effective range, resulting in up to a sevenfold enhancement compared to interaction‐free CAs. Furthermore, the dependence of r_2_ on B_0_ is evaluated across conventional field‐MRI (1.4 and 3.0 T) to UHF‐MRI (7.0 and 11.7 T), in both interacting and non‐interacting CA systems, revealing a nonlinear behavior. These findings establish dipolar interaction control as a key parameter for optimizing T_2_‐CAs performance, advancing the design of next‐generation MRI nanoprobes for diagnostic applications.

## Graphical abstract

In this study, chemically designed nanoscale‐distance‐tuned based on silica‐coated iron oxide nanoparticles are used to investigate how dipolar interactions affect magnetic resonance imaging (MRI) contrast. By varying shell thickness, researchers modulated dipolar interactions and observed an exponential increase in transverse relaxivity (r_2_) that plateaus at higher interaction strengths. Effects are evaluated across conventional and ultra‐high‐field MRI, providing insights for next‐generation T_2_ contrast agents.

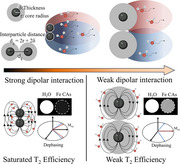

## 1 Introduction

Magnetic resonance imaging (MRI) has long been established as an exceptionally effective modality for the detection and management of various pathologies, playing a crucial role in medical imaging by offering high‐quality, non‐invasive visualization of internal anatomical structures for accurate diagnosis and treatment planning. Its main advantages over other tomographic techniques, such as computed tomography (CT) and positron emission tomography (PET), lie in the absence of ionizing radiation and its ability to provide high‐resolution images.^[^ ^1^, ^2^, ^3^, ^4^ ^]^ However, compared to radioisotope‐based techniques, the low sensitivity of MRI emerges as a significant limitation.^[^ ^5^, ^6^ ^]^ To overcome this barrier, recent advancements in MRI have focused on ultra‐high field (UHF) technologies (B_0_ > 7.0 T), which enhance imaging of complex tissues and allow for more detailed and accurate results.^[^ ^7^, ^8^, ^9^, ^10^ ^]^ UHF‐MRI improves the signal‐to‐noise ratio (SNR) and spatial resolution, enabling enhancements from 0.5 mm (B_0_ = 7.0 T) to 0.2 mm (B_0_ = 9.0 T), facilitating the visualization of microscopic biological structures. Additionally, its validation from a clinical perspective is supported by the Food and Drug Administration (FDA) approval for the use of magnetic fields up to 8.0 T, positioning UHF‐MRI as a promising tool for precise medical diagnostics.^[^ ^11^, ^12^ ^]^

To further enhance MRI sensitivity and accuracy, UHF‐MRI can be combined with advanced contrast agents (CAs).^[^ ^13^, ^14^, ^15^ ^]^ Gadolinium is the most widely used T_1_ CA due to its ability to shorten proton relaxation times and generate positive contrast. However, its clinical use has become increasingly limited due to concerns about toxicity and nephrogenic systemic fibrosis.^[^ ^2^, ^16^ ^]^ Moreover, the longitudinal relaxivity (r_1_) of these CAs decreases drastically at high Larmor frequencies, reducing their effectiveness for T_1_ contrast enhancement in UHF‐MRI.^[^ ^17^, ^18^, ^19^, ^20^ ^]^ In light of these limitations, magnetic nanoparticles (MNPs), particularly iron oxide‐based CAs, have emerged as safer and more versatile alternatives, demonstrating significant performance within the UHF‐MRI regime. While also explored for T_1_ contrast, their main application is as T_2_ agents, since they induce local magnetic field inhomogeneities that accelerate proton dephasing. This influences T_2_ relaxation through spin–spin interactions, reducing signal intensity and generating the negative contrast characteristic of T_2_‐weighted MRI—even at ultra‐high fields.^[^ ^2^, ^21^, ^22^, ^23^ ^]^

Innovative strategies developed in recent years have focused on iron oxide‐based formulations for next‐generation MRI CAs.^[^ ^24^, ^25^ ^]^ While combining T_1_ and T_2_ CAs dates back to 1988,^[^ ^26^ ^]^ technical limitations have led to the development of more effective strategies. Combining iron oxide‐based CAs with T_1_ agents has emerged as a promising dual‐imaging approach, enhancing sensitivity, providing complementary imaging capabilities, and improving tissue differentiation.^[^ ^27^ ^]^ However, optimizing the magnetic properties of iron oxide MNPs remains challenging, particularly in reducing T_2_‐decaying effects while maintaining T_1_ contrast.^[^ ^28^ ^]^

Recent studies have explored a novel distance‐dependent magnetic resonance tuning (MRET) principle, where the separation between a superparamagnetic “quencher” and a paramagnetic “enhancer” precisely modulates T_1_ signal intensity.^[^ ^29^ ^]^ This concept aligns with ongoing efforts to design agents that simultaneously optimize both T_1_ and T_2_ contrast. Achieving a precise interaction between T_1_ and T_2_ components within CAs is crucial for enhancing imaging resolution and controlling signal modulation, thereby improving diagnostic precision.^[^ ^30^ ^]^ Magnetic neutrality can also be achieved via structural defect‐mediated magnetic neutrality nanoprobes (MNNs), synthesized with zinc‐doped ferrite nanoparticles. Structural defects in MNNs minimize T_2_‐decaying effects and enhance T_1_ contrast, particularly for UHF‐MRI.^[^ ^28^ ^]^

Another emerging strategy is the controlled assembly of Fe‐based T_2_ CA clusters, which allows precise modulation of relaxation properties, improving MRI contrast and overall imaging performance.^[^ ^31^, ^32^ ^]^ Several works have highlighted the potential of aggregating MNPs to increase local magnetic field inhomogeneity, focusing on the effect of different design parameters such as the size, shape, and composition of MNP clusters.^[^ ^33^, ^34^, ^35^, ^36^, ^37^, ^38^, ^39^ ^]^ Importantly, the modulation of local magnetic field inhomogeneities plays a dominant role in T_2_ relaxivity, with particle (T_2_‐CA) clustering amplifying these inhomogeneities—a phenomenon further intensified by the incorporation of heterogeneous geometries.^[^ ^32^ ^]^

However, despite extensive research, controlling dipolar interactions remains a major challenge in deepening the understanding of the mechanisms that modulate these inhomogeneities, especially under UHF conditions. Despite recent evaluations of dipolar interaction effects in superparamagnetic nanoparticles,^[^ ^40^ ^]^ their correlation with MRI efficiency remains unexplored, particularly in dispersed nanoparticle systems. This gap is crucial for strategies like nanoparticle clustering, nanocomposites encapsulating T_2_ CAs, and dual T_1_/T_2_ CAs, where precise control of these interactions could optimize performance, maximizing the potential of next‐generation MRI technologies.

**Figure 1:**
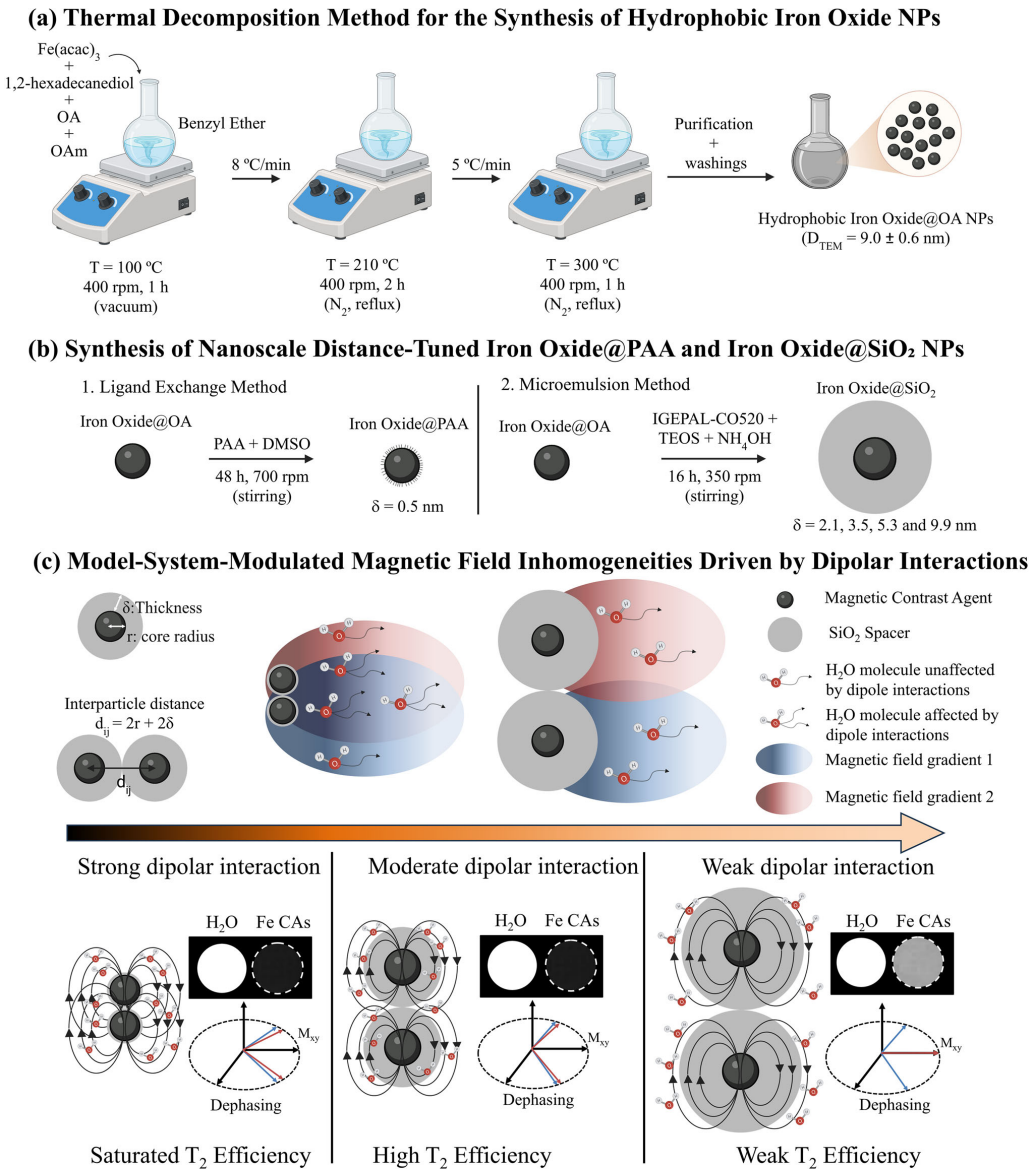
Schematic representation of the proposed nanoscale‐distance‐tuned model system for modulating dipolar interactions in magnetic CAs using non‐magnetic spacers: a) synthetic procedure based on thermal decomposition to obtain the magnetic cores (IO@OA); (b) synthetic route for tuning the shell thickness through ligand exchange (IO@PAA, δ = 0.5 nm) and microemulsion method (IO@SiO_2_, δ = 2.1–9.9 nm); c) interparticle magnetic interactions induce magnetic field inhomogeneities, directly affecting the surrounding water molecules, as modulated by the interparticle distance, d_ij_ = 2r + 2δ (top). Variations in interparticle distance modulate the dipolar regime —from strong to weak—directly affecting dephasing and, consequently, the imaging efficiency of the T_2_ CAs (bottom).

In this study, we designed a nanoscale‐distance‐tuned model system based on 9 nm superparamagnetic iron oxide nanoparticles coated with a non‐magnetic shell of tunable thickness, defined as δ. This design enabled precise control of dipolar interactions by tuning the interparticle spacing, denoted as d_ij_, through δ, thereby affecting local magnetic field inhomogeneities and the interaction of surrounding water molecules (**Figure** **1**). This model allowed the experimental quantification of dipolar interactions on r_2_ via relaxometric measurements and T_2_ MRI maps across clinical (B_0_ < 3.0 T) and UHF‐MRI (B_0_ < 7.0 T) fields, providing, to our knowledge, the first experimental demonstration of the relationship between dipolar interactions and r_2_. Our results show that increasing dipolar coupling initially enhances local magnetic field inhomogeneities and proton dephasing, but its effect follows an exponential trend and plateaus at high dipolar interaction levels, without further improving T_2_ contrast. Conversely, excessive d_ij_ weakens collective effects, limiting contrast modulation. Maximum T_2_ relaxation was achieved under the strongest dipolar interactions (r_2_ = 484 mM^−1^s^−1^), up to seven‐fold higher than the interaction‐free system, highlighting the importance of precise structural control in designing MNP‐based CAs. The role of B_0_ demonstrated a reduction in the efficiency of CAs in the UHF‐MRI regime in environments with interacting MNPs (δ = 0.5 nm). However, a non‐linear increasing trend in r_2_ was observed for interaction‐free systems (δ = 9.9 nm). These findings provide critical insights into the influence of dipolar interactions on proton relaxation dynamics and offer a framework for the novel strategies in the rational design of next generation CAs.

## 2 Results and Discussion

### 2.1 Design and Characterization of the Nanoscale‐Distance‐Tuned T_2_ CAs

A precise nanometric modulation of interparticle distances was achieved by coating the magnetic cores with a non‐magnetic shell of increasing thickness, denoted as δ, serving as spacers to control the magnetic interaction effects between iron oxide MNPs. In this context, δ represents the thickness of the non‐magnetic coating surrounding each magnetic core. Accordingly, d_ij_ can be expressed as: d_ij_ = 2r+2δ, where r is the radius of the magnetic iron oxide core. This relationship highlights that δ directly determines the minimum center‐to‐center distance between two adjacent nanoparticles. This approach induced tunable magnetic field inhomogeneities on water protons, thereby modulating proton dephasing and influencing the efficiency of the CAs in T_2_ MRI (Figure 1).

**Figure 2:**
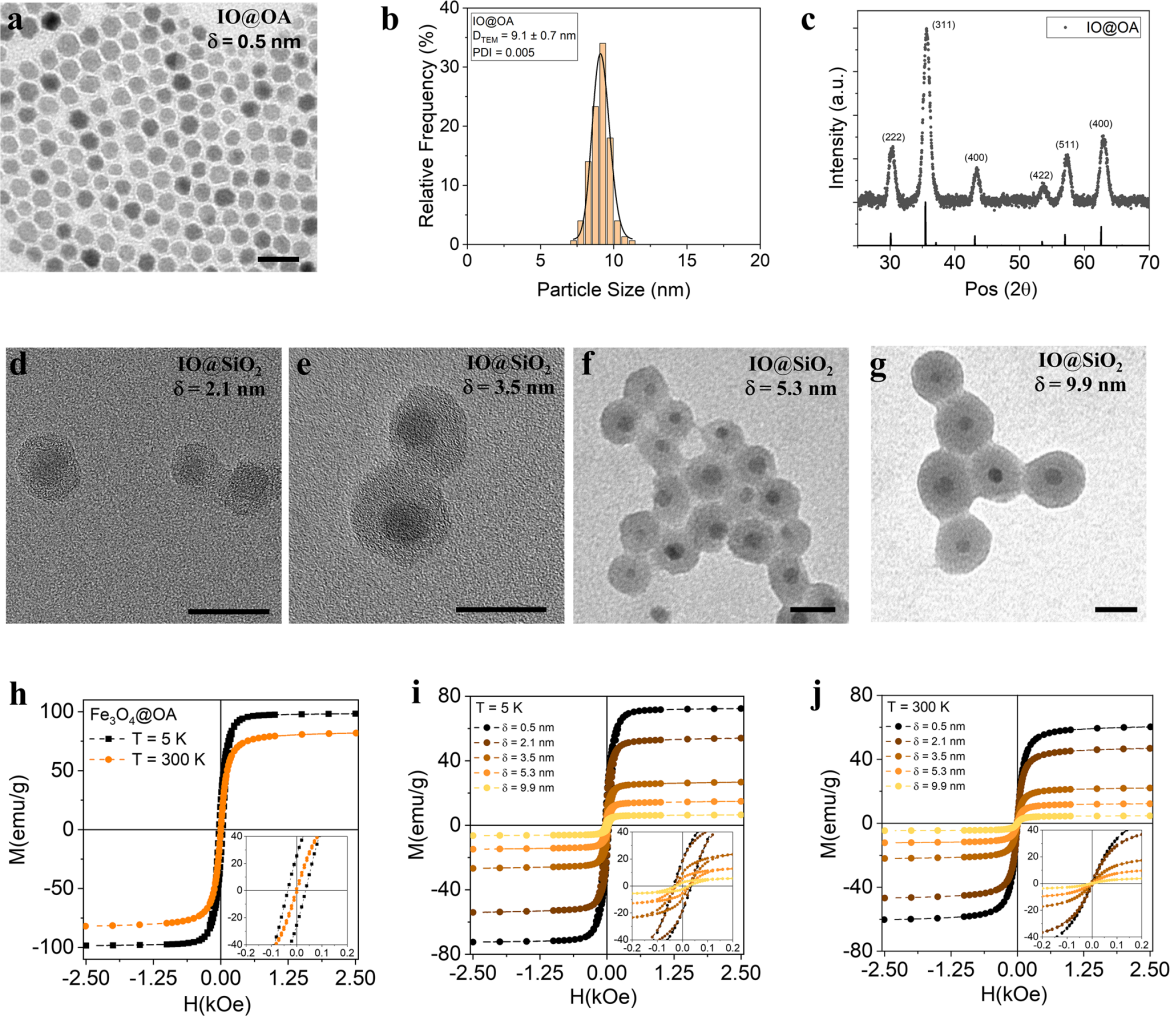
a) Representative TEM micrographs, b) size distribution histograms, and c) X‐ray diffraction patterns of IO@OA MNPs (magnetic core of the nanoscale‐distance‐tuned model system). Representative TEM micrographs of the IO@SiO_2_ MNPs set with different δ: d) δ   = 2.1 nm, e) δ   = 3.5 nm, f) δ   = 5.3 nm, and g) δ   = 9.9 nm. Scale bars of 20 nm. h) Hysteresis loops of IO@OA MNPs at 5 (black) and 300 K (red). The hysteresis loops were normalized to the mass of magnetic material (iron oxide) in the total assembly. Hysteresis loops at i) 5 and j) 300 K of the nanoscale‐distance‐tuned model system with different shell thickness, from dark tones (δ   = 0.5 nm) to light tones (δ   = 9.9 nm). The hysteresis loops were normalized to the total mass. Insets: Scale amplification in hysteresis loops.

The magnetic core of the developed nanoscale‐distance‐tuned model system was composed of superparamagnetic iron oxide MNPs synthesized by thermal decomposition, showing well‐defined spherical morphology and a narrow size distribution (**Figure** **2**a,b, respectively), with an average diameter of 9.1 ± 0.7 nm (PDI = 0.005). The main crystallographic phase identified by the relative position and intensity of the diffraction peaks (Figure 2c) was magnetite/maghemite, consistent with the inverse spinel lattice structure (ICSD card No. 98‐015‐8742).^[^ ^41^ ^]^ As synthesized, MNPs were coated with oleic acid and dispersible in organic solvents. A phase transfer from organic to aqueous medium was carried out using polyacrylic acid (PAA) as capping agent. The obtained IO@PAA MNPs displayed similar morphology and particle size, with D_TEM_ = 8.7 ± 1.2 nm (PDI = 0.02) (Figure S1a, Supporting Information). The control of the interparticle distance was achieved by depositing on the magnetic cores a non‐magnetic SiO_2_ shell of varying thickness via microemulsion method. Transmission electron microscopy (TEM) micrographs of the IO@SiO_2_ MNPs confirmed the successful individual coating of the IO cores and the adjustable SiO_2_ shell thickness, while preserving the spherical morphology after coating (Figure 2d–g). The analysis of the particle size distribution of a series of MNPs coated with SiO_2_ shells of increasing thickness revealed the following average total particle sizes (Figure S1, Supporting Information): D_TEM_ = 13.4 ± 2.2 nm (PDI = 0.03), 16.1 ± 1.9 nm (PDI = 0.01), 19.7 ± 2.3 nm (PDI = 0.01), and 28.8 ± 2.6 nm (PDI = 0.008). The shell thickness in the samples was δ  = 2.2, 3.5, 5.3, and 9.9, respectively (**Table** **1**).

**Table 1:** Average nanoparticle sizes obtained from TEM micrographs analysis (D_TEM_) and their corresponding PDI_TEM_; R_core/shell_, defined as the ratio of core to shell mass fractions, determined from ICP and TGA analyses; hydrodynamic sizes (D_H_) and their corresponding PDI_DLS_, acquired through DLS; and ζ‐potential values of MNPs.

Sample	D_TEM_ [nm]	δ [nm]	PDI_TEM_	R_core/shell_	D_H_ [nm]	PDI_DLS_	ζ‐Potential [mV]
IO@OA – 0.5 nm	9.1 ± 0.6	0.5	0.005	2.8	—	—	—
IO@PAA – 0.5 nm	8.6 ± 1.2	0.5	0.02	3.5	63.7 ± 22.9	0.13	−36.3
IO@SiO_2_ – 2.1 nm	13.4 ± 2.2	2.1	0.03	0.7	124.8 ± 30.5	0.06	−44.0
IO@SiO_2_ – 3.5 nm	16.1 ± 1.9	3.5	0.01	0.5	79.4 ± 23.8	0.09	−48.2
IO@SiO_2_ – 5.3 nm	19.7 ± 2.3	5.3	0.01	0.1	75.5 ± 25.1	0.11	−54.3
IO@SiO_2_ – 9.9 nm	28.8 ± 2.6	9.9	0.008	0.05	54.3 ± 17.3	0.10	−60.3

Inductively coupled plasma emission spectroscopy (ICP) and thermogravimetric analysis (TGA) were performed to determine the Fe content of the core and the mass fraction of the shell, respectively, enabling the calculation of R_core/shell_ = mass of core/mass of shell (Table 1). MNPs coated with thin organic shells (OA, PAA, ≈0.5 nm) exhibited higher R_core/shell_ values (2.8 and 3.5, respectively), whereas progressively thicker SiO_2_ shells (2–10 nm) led to a substantial reduction of the ratio, from 0.7 to 0.05, reflecting the increasing dominance of the shell contribution in the overall mass distribution. These values were consistent with the δ measured by TEM, confirming that R_core/shell_ decreased as δ grew, and that both ICP/TGA and TEM data consistently reflected the progressive increase of the shell contribution to the total particle mass.

XRD analysis confirmed that the nanometrically controlled growth of the shell does not alter the primary crystallographic phase of the precursor magnetic core, which remains magnetite/maghemite with the characteristic inverse spinel structure (Figure S2, Supporting Information). Importantly, no diffraction peaks corresponding to secondary phases or impurities were detected, thereby confirming the structural and compositional purity of the samples. For samples with very high SiO_2_ content (δ = 5.3 and 9.9 nm), a broad feature at 2θ ≈ 22° is observed, corresponding to amorphous SiO_2_, which diminishes as the R_core/shell_ increases. FTIR spectroscopy confirmed the surface functionalization of the nanoparticle model system (Figure S3, Supporting Information). The Fe─O stretching (≈580 cm^−1^), associated with the tetrahedral Fe^3^⁺–O^2^^−^ groups, was observed in all samples, confirming the presence of the magnetite/maghemite cores.^[^ ^42^ ^]^ In precursor IO@OA MNPs, C─H (≈2920, 2850 cm^−1^) and C═O/COO^−^ (≈1700, 1400 cm^−1^) bands indicated oleic acid binding to the surface.^[^ ^43^ ^]^ IO@PAA MNPs, derived from IO@OA, showed asymmetric (≈1600 cm^−1^) and symmetric (≈1400 cm^−1^) COO^−^, C─O (≈1100–1300 cm^−1^), and broad O─H (≈3200–3400 cm^−1^) bands, consistent with PAA functionalization, indicating successful surface modification and increased hydrophilicity.^[^ ^44^ ^]^ In IO@SiO_2_ MNPs, the Fe─O band progressively decreased with increasing δ, while Si─O─Si asymmetric stretching (≈1100–1000 cm^−1^), Si─O─Si symmetric stretching (≈800 cm^−1^), and Si─O bending (≈460 cm^−1^) confirmed the formation of a uniform silica shell over the magnetic cores.^[^ ^45^ ^]^

Magnetic behavior plays a key role in T_2_ CAs, not only through saturation magnetization (M_S_), which directly modulates r_2_, but also via the modification of the collective behavior of the MNPs, influenced by parameters such as remanence (M_R_) and coercivity (H_C_). The precursor magnetic core (IO@OA), which is the only magnetic component of the system, exhibited values of M_S_ = 82 (99) emu·g^−1^, M_R_ = 1 (25) emu·g^−1^ and H_C_ = 21 (356) Oe at 300 (5) K, as measured from the hysteresis loops normalized to magnetic mass (Figure 2h). Similarly, hysteresis loops were obtained for the nanoscale‐distance‐tuned model samples with varying δ, at 5 and 300 K respectively (Figure 2i,j). At room temperature, M_S_ values ranged from 61 emu·g^−1^ (δ  = 0.5 nm) to 5 emu·g^−1^ (δ  = 9.9 nm), indicating a decrease in total magnetization due to the increasing contribution of non‐magnetic mass to the total mass with silica shell. Similar H_C_ values were obtained across the whole SiO_2_‐coated series, H_C_ = 35 Oe, slightly higher than that of IO@OA (21 Oe). The M_R_ values were close to zero, with a maximum value of 2 emu·g^−1^ (δ  = 2.1) and a minimum value of 0.1 emu·g^−1^ (δ = 9.9 nm). At 5 K, the M_S_ values increased, with a maximum value of 73 emu·g^−1^ (δ  = 0.5 nm) and a minimum value of 5 emu·g^−1^ (δ  = 9.9 nm). The H_C_ and M_R_ values also increased, with maximum values of 356 Oe and 19 emu·g^−1^ (δ  = 0.5 nm) and minimum values of 262 Oe and 2 emu·g^−1^ (δ  = 9.9 nm), respectively, suggesting a magnetically blocked behavior at 5K, as expected.

The colloidal stability of MNPs in aqueous media is key to evaluate their efficiency as CAs. These properties were evaluated through dynamic light scattering (DLS) measurements, by the determination of both D_H_ and ζ‐potential (Figure S4, Supporting Information; Table 1). Although TEM shows that the silica shell thickness increases around the 9 nm magnetic cores, D_H_ decreases with increasing δ: 124.8 nm (δ = 2.1 nm), 79.4 nm (δ = 3.5 nm), 75.5 nm (δ = 5.3 nm), and 54.3 nm (δ = 9.9 nm). This trend suggests that thinner shells are associated with larger D_H_, likely reflecting partial aggregation within the SiO_2_‐coated MNP series. This phenomenon suggests that reducing δ leads to stronger dipolar interactions, which in turn promote increased aggregation of the nanoparticles. Polydispersity index (PDI) values remain below 0.2, indicating a generally homogeneous dispersion with low polydispersity. The ζ‐potential was significantly negative across the whole series (i.e., −60.3 mV for the IO@SiO_2_ – 9.9 nm sample), indicating strong electrostatic repulsion and further confirming high water colloidal stability of the MNPs dispersions.

### 2.2 MRI Characterization of the Nanoscale‐Distance‐Tuned T _2_ CA System

**Figure 3:**
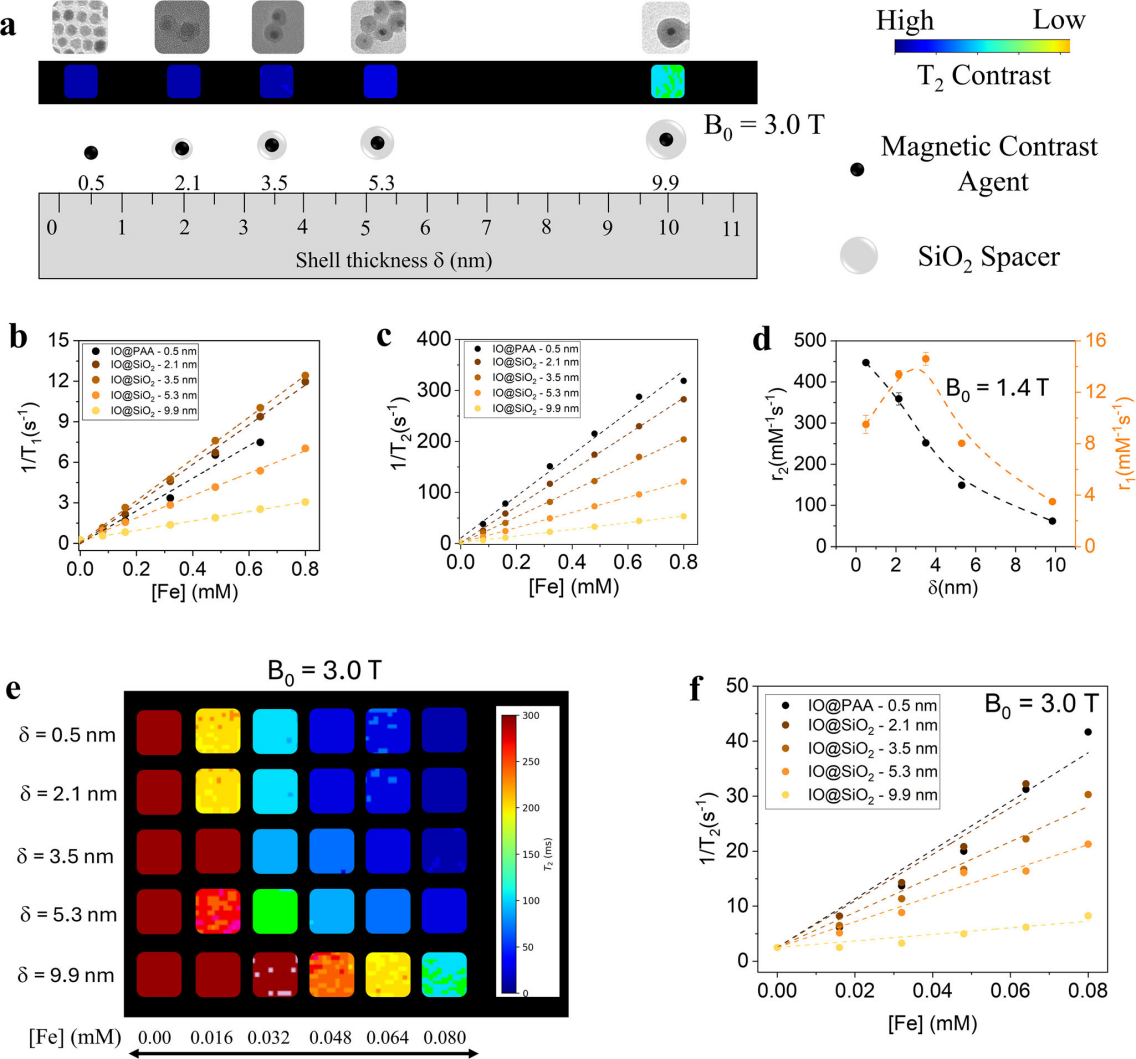
a) Schematic representation of the nanoscale‐distance‐tuned model system with variable thickness shell on a nanometer scale: Representative TEM image of MNPs corresponding to each δ and their associated MRI T_2_ signals (B_0_ = 3.0 T, 0.08 mM), modulated through SiO_2_‐based spacers (gray shell) of the CAs (black spheres). b) 1/T_1_ and c) 1/T_2_ as a function of Fe concentration (0.00–0.80 mm) for MNPs with different δ, from δ = 0.5 nm (dark tones) to δ = 9.9 nm (light tones). d) r_2_ (black) and r_1_ (red) values obtained from the slope of the 1/T_1,2_ versus [Fe] fit, as a function of δ. e) T_2_ MRI map acquired at a B_0_ = 3.0 T, ranging from δ = 0.5 nm (top) to δ = 9.9 nm (bottom) at different Fe concentrations up to 0.08 mm. f) Transverse relaxation time, 1/T_2_ as a function of [Fe] up to 0.08 mM, from δ  = 0.5 nm (dark tone) to δ = 9.9 nm (light tone), obtained from the T_2_ MRI maps.

The variation of the particle‐particle nanoscale distance modulates the MRI signal and the efficiency of the MNPs as CAs. At the same Fe concentration, T_2_ signal decreases as the magnetic cores are separated by increasing δ (**Figure** **3**a). This distance‐dependent modulation was carefully quantified using various techniques. Relaxation times were initially measured using relaxometric methods (B_0_ = 1.41 T), and the relaxivities r_1_ and r_2_ for each MNP were determined from the slope of the relaxation rate 1/T_1,2_ versus Fe concentration plots (Figure 3b,c). Interestingly, while r_1_ exhibits a maximum at δ = 3.5 nm, r_2_ monotonically decreases as δ increases (Figure 3c), as expected due to the larger distance the water molecules are from the magnetic cores. r_2_ values ranged from 446 ± 4 mM^−1^·s^−1^ (δ = 0.5 nm) to 62 ± 3 mM^−1^·s^−1^ (δ = 9.9 nm), showing that δ (or d_ij_) effectively tunes T_2_‐MRI performance. Previous relaxivity studies on silica‐coated iron oxide MNPs highlight the importance of water permeability in SiO_2_ shells, showing that for small particles (≤28 nm) the silica layer is largely impermeable, while a water‐permeable fraction appears for particles >40 nm, playing an important role in the modulation of both r_1_ and r_2._^[^ ^45^, ^46^, ^47^, ^48^ ^]^ In our system, the shells are thin (0.5–9.9 nm), well below the thickness at which a significant permeable fraction would develop and fall within the regime where dipolar interactions become relevant. This impermeability in thin shells could account for the observed differences in r_1_ trends between our system and previous reports, where r_1_ decreased monotonically with increasing shell thickness.

To further confirm the relaxivity data and translate the numerical results into meaningful MR signals, phantom images of the samples were acquired at a clinically field strength. First, a T_2_ signal variation analysis was performed at B_0_ = 3 T (Figure S5a, Supporting Information). The signal intensity of increasing CA concentrations at different shell thicknesses was measured and normalized to pure H_2_O. The variation in signal intensity (ΔIntensity) increased with concentration (up to 0.08 mM Fe) and decreased with δ, from −93.58% (δ = 0.5 nm, IO@PAA – 0.5 nm) to ‐38.5% (δ = 9.9 nm, IO@SiO_2_ – 9.9 nm) at the same concentration, reflecting the effect of the non‐magnetic spacer (Figure S5b, Supporting Information). A T_2_ map was also obtained at B_0_ = 3 T across 0–0.08 mM Fe, showing shorter T_2_ times at higher concentrations and longer T_2_ times as δ increases, leading to poorer contrast (Figure 3e). r_2_ values were determined from the slope of 1/T_2_ versus Fe concentration (Figure 3f and **Table** **2**). Consistent with previous relaxivity measurements, r_2_ decreased with increasing δ, from a maximum of 476 ± 48 mM^−1^·s^−1^ (δ = 0.5 nm) to a minimum of 74 ± 10 mM^−1^·s^−1^ (δ = 9.9 nm) at B_0_ = 3 T.

**Table 2:** r_2_ values obtained by the relaxometry measurements (B_0_ = 1.4 T) and by the MR images (B_0_ = 3.0, 7.0 and 11.7 T).

Sample	r_2_ [mM^−1^ s^−1^]			
	B_0_ = 1.4 T	B_0_ = 3.0 T	B_0_ = 7.0 T	B_0_ = 11.7 T
IO@PAA – 0.5 nm	447 ± 6	476 ± 48	473 ± 7	455 ± 11
IO@SiO_2_ – 2.1 nm	359 ± 15	450 ± 40	403 ± 4	406 ± 4
IO@SiO_2_ – 3.5 nm	252 ± 3	341 ± 20	227 ± 1	243 ± 1
IO@SiO_2_ – 5.3 nm	149 ± 1	241 ± 22	183 ± 1	200 ± 2
IO@SiO_2_ – 9.9 nm	62 ± 3	74 ± 10	74.3 ± 1	84 ± 1

Understanding the effects of magnetic field strength on the efficiency of CAs under UHF‐MRI conditions is increasingly relevant due to recent FDA approvals. Although a direct correlation between B_0_ and r_2_ may not exist, variations in the M_S_ of MNPs, which are directly associated with B_0_, notably affect r_2_.^[^ ^49^, ^50^ ^]^ Conventionally, the performance of T_2_‐CAs remains largely unaffected at high field strengths, primarily because magnetic saturation is quickly reached (Figure 2h). However, the effect of B_0_ on magnetic dipolar interactions does not necessarily need to follow the same trend.

**Figure 4:**
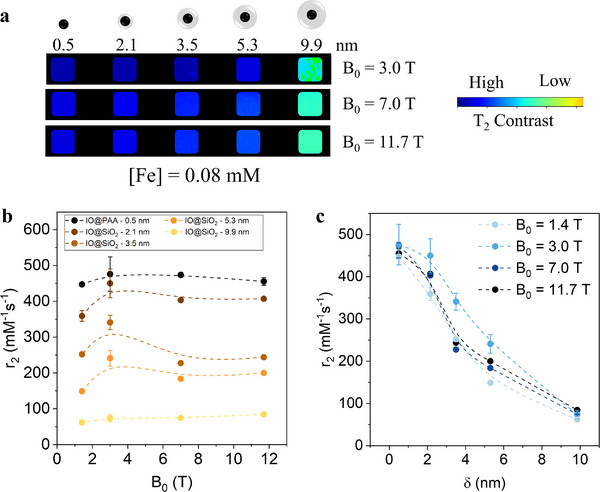
a) T_2_‐MRI signal of the nanoscale‐distance tuned model system at B_0_ = 3.0, 7.0 and 11.7 T ([Fe] = 0.08 mM) − SiO_2_ spacer (gray shell), iron oxide CAs (black spheres). Dependency of r_2_ b) on the magnetic field strength for each shell thickness sample from δ = 0.5 nm (dark tones) to 9.9 nm (light tones) and c) on δ at different field strengths, from B_0_ = 1.4 T (light tones) to B_0_ = 11.7 T (dark tones).

To investigate the effect of the nanoscale separation between T_2_ CAs in the UHF‐MRI regime, the r_2_ values of the nanoscale‐distance‐tuned model system were evaluated from T_2_ maps acquired at B_0_ = 7 T (Figure S6, Supporting Information) and B_0_ = 11.7 T (Figure S7, Supporting Information). A comprehensive analysis of field strength effects on r_2_ across samples with varying δ was performed using the field‐dependent r_2_ data (**Figure** **4**a). As observed at lower fields, increasing δ significantly reduces r_2_ in the UHF‐MRI regime. Generally, r_2_ initially increases from B_0_ = 1.4 T to 3.0 T, particularly at small δ (Figure 4b), which can be ascribed to progressive particle spin alignment and increased M_S_. Further increase from B_0_ = 7.0 T to 11.7 T produces minor changes in r_2_, with small decreases for δ = 3.5 and 5.3 nm, while the thickest shell (δ = 9.9 nm) shows the greatest stability. These observed trends might be attributed to various phenomena. Initially, the significant augmentation at low B_0_ could be attributed to increased magnetization of the MNPs. Conversely, at higher fields (B_0_ = 7.0 T and B_0_ = 11.7 T), this effect diminishes as the MNPs reach M_S_, as evidenced in the hysteresis loops. At UHF‐MRI, reduced r_2_ may arise from stronger magnetic interactions, local field fluctuations, anisotropy changes, or particle aggregation, whereas thicker shells mitigate these effects by increasing interparticle distance and reducing dipolar interactions. The effect of interparticle distance on r_2_ is more clearly manifested in Figure 4c, with r_2_ decreasing from 450 mM^−1^·s^−1^ (δ = 0.5 nm) to 70 mM^−1^·s^−1^ (δ = 9.9 nm).

This nanoscale distance‐dependent effect has already been introduced in recent studies and has been attributed to the influence of the spacer coating, which modulates the distance between the CAs and the neighboring water protons.^[^ ^51^, ^52^ ^]^ A dependence of R_2_ (1/T_2_) on the spacer coating has been modelled, suggesting that its structural and chemical properties modulate the degree of water accessibility to the magnetic core—either by restricting water diffusion or by increasing residence time through hydrogen bonding:^[^ ^53^ ^]^

(1) $$R_{2}=\frac{\left(\frac{256\pi^{2}\gamma_{H}^{2}}{405}\right)V^{*}{\left(M_{S}a\right)}^{2}}{D\left(1+\frac{\delta }{a}\right)}$$

where V* represents the volume fraction of the magnetic core, M_S_ stands for the saturation magnetization of the nanoparticles, D is the diffusion coefficient of water molecules, a denotes the radius of the core, and δ indicates the thickness of an impermeable surface coating. Although experimental results from the nanoscale‐distance‐tuned model demonstrate a clear decrease in r_2_ with increasing spacer thickness, this deviates from the predictions of Equation (1). This discrepancy suggests the presence of additional mechanisms. Dipolar interactions, known to dominate in nanoscale distance‐dependent systems,^[^ ^40^ ^]^ could emerge as a significant factor, influencing water molecule relaxation dynamics through changes in the field inhomogeneities generated by the CAs, potentially explaining the observed deviations.

### 2.3 Quantification of Dipolar Interactions in the Nanoscale‐Distance‐Tuned T_2_ CAs

To better understand the factors influencing imaging efficiency through r_2_, it is crucial to investigate interparticle dipolar interactions within the nanoscale‐distance‐tuned model system. Considering each particle as a single magnetic domain, in which all atomic moments rotate coherently, the total magnetic moment can be expressed as $\left|{\vec{\mu }}_{i}\right|=M_{s}\cdot V_{i}$, where V_i_ is the particle volume and M_S_ is the saturation magnetization. Based on this well‐established model, the magnetic field generated by each particle affects neighboring moments, giving rise to dipole–dipole interactions. The magnetic dipolar interaction between two particles i and j, separated by the interparticle distance, ${\vec{d}}_{\mathrm{ij}}$, is given by:^[^ ^54^ ^]^

(2) $$E_{D}^{\left(i,j\right)}=\frac{{\vec{\mu }}_{i}\cdot {\vec{\mu }}_{j}}{d_{\mathrm{ij}}^{3}}-\frac{3\left({\vec{\mu }}_{i}\cdot {\vec{d}}_{\mathrm{ij}}\right)\left({\vec{\mu }}_{j}\cdot {\vec{d}}_{\mathrm{ij}}\right)}{d_{\mathrm{ij}}^{5}}$$

This energy depends strongly on the relative orientation of the dipoles and d_ij_, decaying rapidly with the cube of separation. Consequently, small variations in particle spacing, such as those introduced by SiO_2_ coatings of varying thickness, can significantly modify E_D_ and r_2_. These interactions should be examined in colloidal suspensions rather than only in the powdered state—where they are commonly studied due to technical challenges— to better reflect conditions relevant to MRI. To rigorously probe these dipolar effects under physiologically relevant conditions, a thorough approach was implemented, combining two methodologies: DC magnetometry (zero‐field‐cooled and field‐cooled—ZFC‐FC—curves and Henkel plots) and AC magnetometry (Vogel‐Fulcher law applied to characterize dynamic magnetic behaviours).

**Figure 5:**
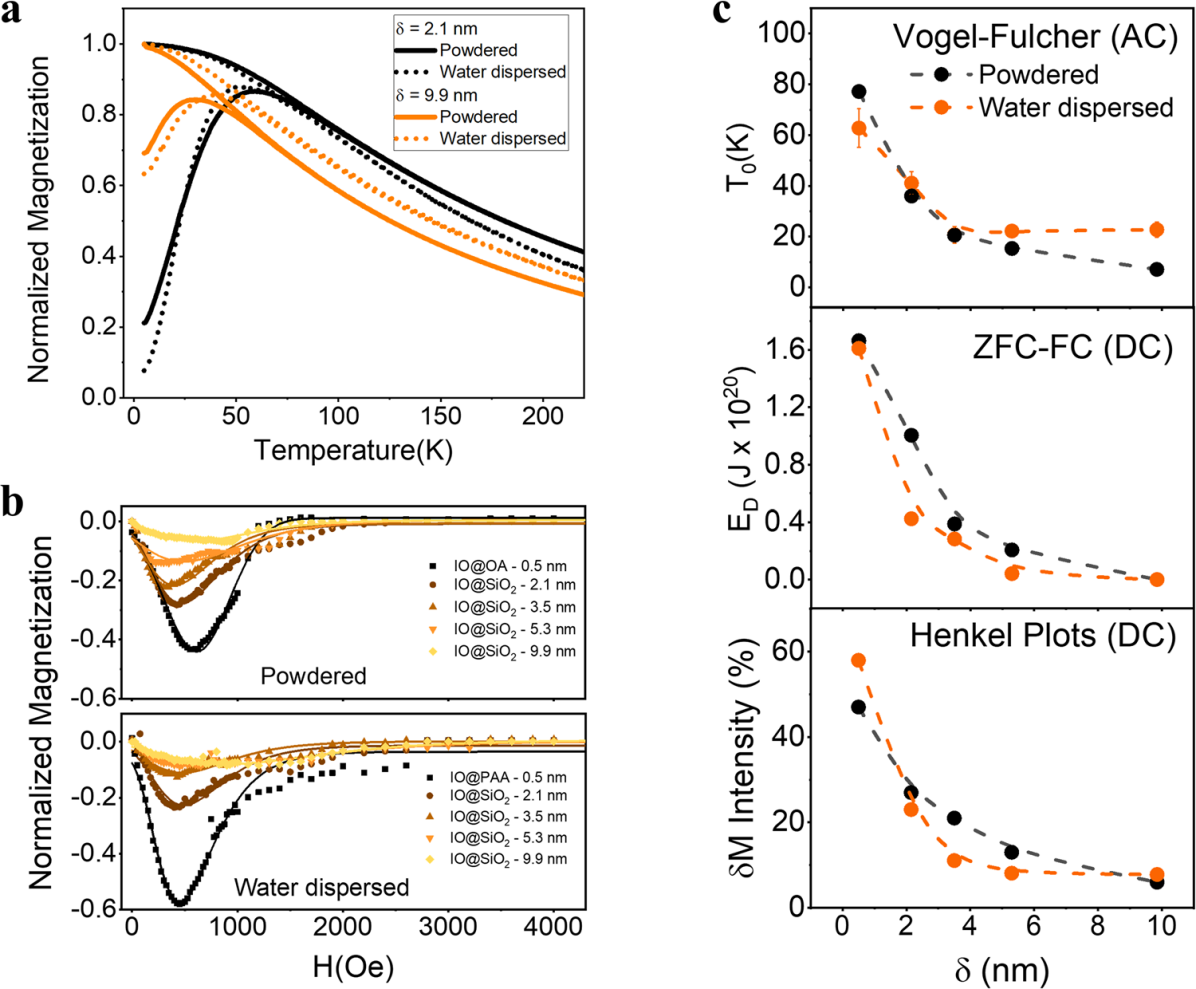
a) ZFC‐FC curves of two representative silica‐coated MNPs, δ  = 2.1 nm (black) and δ  = 9.9 nm (orange), in powder (solid line) and aqueous‐dispersed (dotted line) states. b) δM normalized curves of powder (top) and aqueous‐dispersed (0.8 mM, bottom) MNPs with different silica shell thicknesses: from dark tones (δ  = 0.5 nm) to light tones (δ = 9.9 nm). c) Interaction values obtained through different procedures: from the Vogel‐Fulcher law (top), using ZFC‐FC protocols (middle), and through Henkel plots (bottom). Black and orange dots represent the different states of the samples: powder and dispersed in aqueous media, respectively.

Starting with DC magnetometry, the temperature dependence of magnetization was measured under ZFC and FC conditions in both powdered and aqueous‐dispersed MNPs at a Fe concentration of 0.8 mm (Figure S8, Supporting Information). The comparison of the ZFC‐FC curves of the samples with thinner (δ = 2.1 nm, black curves) and thicker (δ = 9.9 nm, orange curves) SiO_2_ shells in both powder and water dispersible states is shown in **Figure** **5**a. The blocking temperature, T_B_—the temperature where thermal energy equals the anisotropy energy barrier, modulated by dipolar interactions—was determined from the ZFC maxima, showing a clear dependence on δ. Across the full δ range, T_B_ decreases with increasing δ, following a d_ij_^−^^3^ decay, from T_B_ = 78.5 and 86.9 K (δ = 0.5 nm) to T_B_ = 30.3 and 40.2 K (δ = 9.9 nm) for powdered and aqueous‐dispersed MNPs, respectively (Figure S8, Supporting Information). This decrease reflects the reduction in E_D_ with larger d_ij_, consistent with previous powder‐based studies.^[^ ^40^ ^]^ The similarity between aqueous‐dispersed and powder trends indicates that dipolar interactions remain significant in dispersed samples, affecting magnetic properties and imaging efficiency. Measurements at different Fe concentrations (0.16–0.80 mm, Figure S9, Supporting Information) showed no clear trend in T_B_, suggesting minor influence of concentration due to non‐uniform distribution or aggregation, while SiO_2_ thickness remains the primary factor controlling dipolar interactions.

To more precisely demonstrate the role of dipolar interactions, E_D_ was obtained based on the Arrhenius‐Néel law, following a previously described model that incorporates an E_D_ term in the energy barrier, ΔE (described in Supporting Information).^[^ ^54^ ^]^ The dependence of r_2_ on E_D_ shows a clear decrease with increasing δ (Figure 5c). The maximum E_D_ (1.6·10^2^⁰ J) occurs at the minimum δ = 0.5 nm for both powdered and water‐dispersed MNPs, and decreases as δ increases, reaching values of E_D_ = 0.42·10^20^, 0.28·10^20^, 0.04·10^20^, and 0 J for δ  = 2.1, 3.5, 5.3, and 9.9 nm, respectively, for the water‐dispersed MNPs.

Henkel plots, denoted as δM, were employed as an alternative method to directly evaluate the effects of interparticle interactions. This approach provides a more precise understanding of how these interactions influence the magnetic properties of the CAs, providing insights complementary to other measurement techniques (described in Supporting Information). The representative δM curves for the entire nanoscale‐distance‐tuned model system reveal a negative peak, corresponding to the minimum of δM and indicative of prevalent dipolar interactions (Figure 5b). This is in stark contrast to the absence of a peak (indicating negligible interactions) or a positive peak (suggesting exchange interactions). The intensity dependence of δM for the nanoscale‐distance‐tuned T_2_ CAs system (Figure 5c) showcases a consistent decrease of dipolar interactions with increasing δ, both in powdered (black dots) and aqueous‐dispersed state (orange dots). The δM peak intensity, reflecting the strength of dipolar interactions, decreases consistently with increasing δ for both powdered (black) and aqueous‐dispersed (orange) states (Figure 5c), ranging from 47.5% and 57.9% (δ = 0.5 nm) to 5.2% and 7.8% (δ = 9.9 nm), confirming that larger δ reduces dipolar interactions. In liquid media, δM decreases more abruptly, flattening from δ = 5.3 nm and reaching 8.1%, close to the minimum of 7.8% at δ = 9.9 nm.

AC magnetometry was then employed to corroborate these results, providing an additional layer of verification by assessing the dynamic magnetic properties of the MNPs. The temperature dependence of AC susceptibility (χ') was evaluated over a frequency range of 50–750 Hz for MNPs in both powdered and aqueous‐dispersed states, with δ ranging from 0.5 to 9.9 nm (Figures S10 and S11, Supporting Information). From these measurements, interaction effects were quantified using the Vogel‐Fulcher law, which is based on relaxation effects (Figures S12 and S13 (Supporting Information), detailed in the Supporting Information). The interaction temperature parameter, T_0_, which quantifies the strength of dipolar interactions—higher values indicate stronger interactions, while values approaching zero correspond to negligible interactions—was found to decrease consistently with increasing δ (Figure 5c). For powdered MNPs, T_0_ decreased from 77 K (δ = 0.5 nm) to 7 K (δ = 9.9 nm), while for aqueous‐dispersed MNPs it ranged from 69 to 21 K. Interestingly, for aqueous‐dispersed MNPs with δ ≥ 3.5 nm, T_0_ exhibited minimal changes, reaching 19 K at δ = 9.9 nm. This behavior suggests that, while dipolar interactions remain significant in aqueous dispersions, their modulation becomes less sensitive to shell thickness beyond certain thresholds.

### 2.4 Role of Dipolar Interactions in the MRI Efficiency of the Nanoscale‐Distance‐Tuned T_2_ CAs

**Figure 6:**
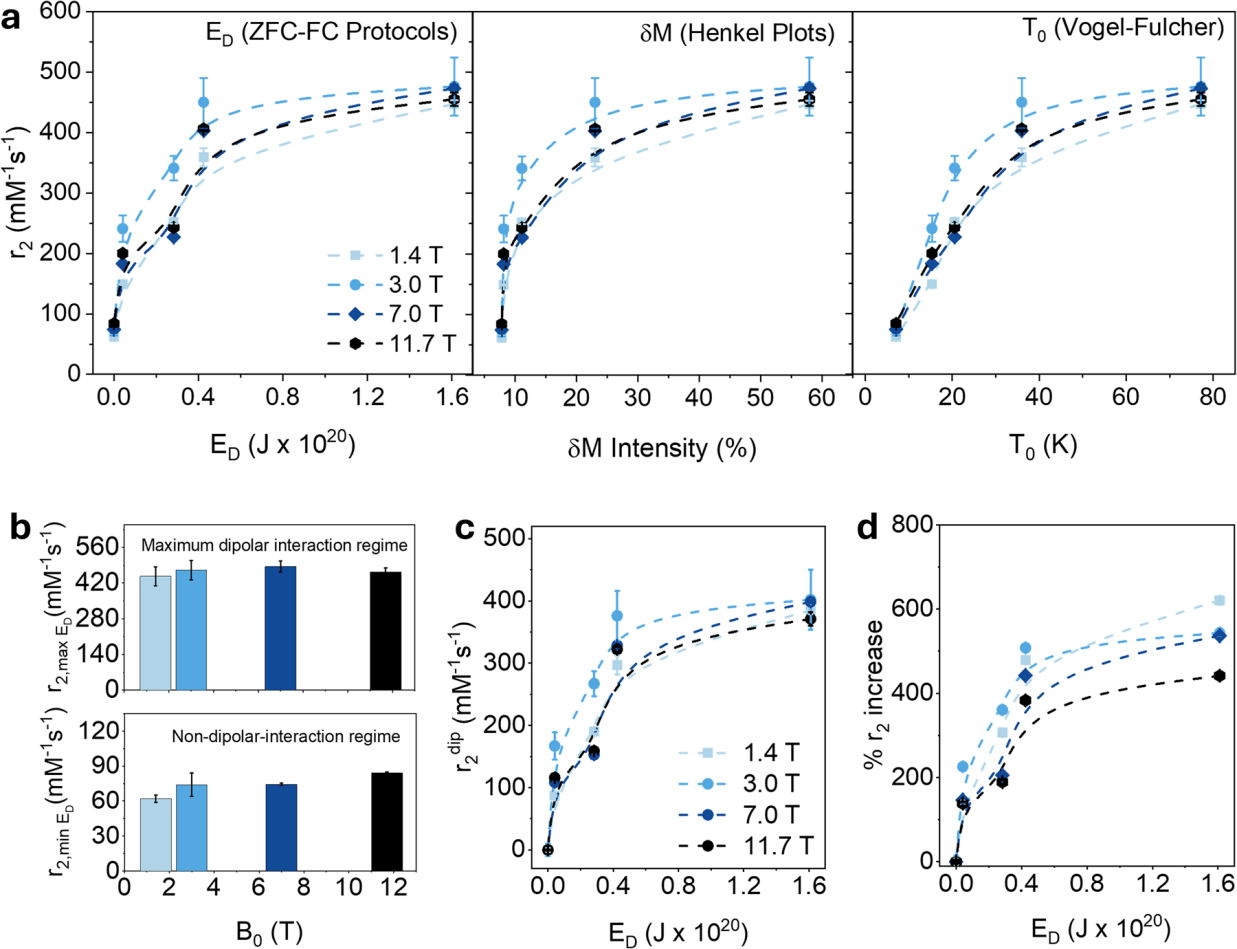
a) Dependence of r_2_ on dipolar interactions: Dipolar energy obtained through ZFC‐FC protocols, Henkel Plot intensity derived from DC Magnetometry, and T_0_ parameter obtained by AC magnetometry via the Vogel–Fulcher law. Measurements were performed with MNPs dispersed in water. b) Relaxivity values free of interaction and coating effects (r_2,min ED_) and in the maximun dipolar interaction regime (r_2,max ED_) as a function of the magnetic field. c) Relaxivity values attributed to dipolar interaction, r_2_^dip^, and d) percentage increase in r_2_ as a function of the applied dipolar energy (E_D_).

The nanometric‐scale modulation of the distance between CAs was found to produce a strong effect on dipolar interactions, as confirmed by three independent analytical methods (Figure 5c). A clear correlation was observed: smaller δ leads to stronger dipolar interactions, which remain significant even in aqueous dispersed CAs. This persistent interaction may contribute to the observed decrease in the r_2_ with increasing δ (Figure 4c). To systematically investigate the effect of dipolar interactions on T_2_ CA efficiency, r_2_ was analyzed as a function of the dipolar energy (E_D_, obtained from ZFC‐FC protocols), the δM peak intensity from Henkel plots, and the interaction temperature parameter T_0_ (derived from the Vogel–Fulcher law) (**Figure** **6**a). As dipolar interactions increased (and δ and d_ij_ decreased), r_2_ rises until a plateau is reached for δ < 2.5 nm. This plateau, observed at E_D_ ≈ 0.42·10^2^⁰ J (corresponding to a dipolar interaction intensity of 24 %), indicates that excessively strong dipolar interactions do not further enhance r_2_, revealing a saturation regime, which can be further analyzed quantitatively.

To quantify this behavior, r_2_ was fitted as a function of E_D_ using an exponential decay model, r_2_(x) = a⋅e^bx^+c (Figure S14, Supporting Information). In all cases, a good fitting was observed and a negative value for the parameter “b” indicates an exponential decrease of r_2_ with increasing dipolar interaction energy. This behavior suggests a phase‐like transition between distinct relaxation regimes: from the motional averaging regime (MAR), characterized by a positive correlation between r_2_ and interaction strength, to the static dephasing regime (SDR), where relaxivity tends to saturate, and even toward the echo limiting regime (ELR), where r_2_ may even decrease due to excessive local field inhomogeneities.

From this exponential fit, the intrinsic relaxivity unaffected by dipolar interactions (r_2,min ED_), corresponding to the maximum interparticle distance (δ = 9.9 nm), was estimated. These values, obtained for the different magnetic fields strengths used, were found to be r_2,min ED_ = 62 mM^−1^s^−1^ (B_0_ = 1.4 T), 74 mM^−1^s^−1^ (B_0_ = 3.0 T), 74 mM^−1^s^−1^ (B_0_ = 7.0 T), and 84 mM^−1^s^−1^ (B_0_ = 11.7 T) demonstrating a positive effect of B_0_ on r_2_ (Figure 6b). This finding represents, to our knowledge, the first experimental demonstration of the r_2_ dependence on B_0_ for a T_2_ CAs under ideal conditions (dipolar interaction‐free conditions). It highlights the critical role of dipolar interactions, which intensify at higher magnetic fields in interacting CA systems, leading to enhanced r_2_ values. On the other hand, the r_2_ under maximum dipolar interactions (δ = 0.5 nm, d_ij_ minima), denoted as r_2,max ED_ was obtained from the asymptotic limit (x → ∞) of the exponential fit (E_D_ vs r_2_), revealing values of r_2,max ED_ = 447 mM^−1^s^−1^ (B_0_ = 1.4 T), 471 mM^−1^s^−1^ (B_0_ = 3.0 T), 485 mM^−1^s^−1^ (B_0_ = 7.0 T), and 464 mM^−1^s^−1^ (B_0_ = 11.7 T). Trends in the maximum dipolar interaction regime differ slightly from the interaction‐free case, with a small decrease in r_2_ observed under UHF‐MRI conditions (Figure 6b).

The r_2_ modulation induced by dipolar interactions (r_2,dip_, representing the relaxivity contribution solely due to dipolar interactions) was quantified as a function of E_D_ by subtracting the interaction‐free relaxivity (r_2,min_) from the measured r_2_ values (Figure 6c). In an intermediate state of dipolar interactions (E_D_ = 0.28·10^20^ J, δ = 2.5 nm), the T_2_ CAs reached near‐maximal efficiency, with r_2_^dip^ values exceeding 300 mM^−1^s^−1^. A slight increase was still observed as the system approached the regime of maximum dipolar interaction (E_D_ = 0.40·10^20^ J, δ = 0.5 nm). However, the rate of increase slowed considerably, indicating that the contribution of dipolar interactions becomes drastically reduced. The percentage increase in r_2_ relative to the dipolar interaction‐free state, calculated as ((r_2_ – r_2,min_)/r_2,min_)·100, reached nearly 600 %, corresponding to a sevenfold enhancement in CA efficiency compared to the interaction‐free condition (Figure 6d).

These results indicate that the observed reduction in r_2_ at high B_0_ does not reflect an intrinsic limitation of MRI in the UHF‐MRI regime, but arises from local magnetic field inhomogeneities induced by dipolar interactions. Stronger dipolar interactions could generate an additional local field, B_0_′, dependent on d_ij_ and relative orientation, which perturbs the homogeneous B_0_ field. This perturbation modifies the local Larmor frequency (ω′ = γ(B_0_ + B_0_′)) and, in the low‐interaction regime, enhances transverse spin dephasing, consequently increasing r_2_. Overall, these findings highlight the need to balance magnetic field strength and dipolar interactions to optimize iron oxide‐based T_2_ CA performance, emphasizing the importance of rational design strategies for next‐generation CAs.

## 3 Conclusion

In this work, we developed a nanoscale‐distance‐tuned T_2_ CA model system based on iron oxide MNPs coated with non‐magnetic SiO_2_ layers of variable thickness. By precisely controlling the minimal interparticle distance at the nanoscale, we systematically modulated dipolar interactions and evaluated their impact on r_2_ and MRI contrast efficiency. Relaxometry and MR imaging were performed at clinical and ultra‐high field strengths (B_0_ = 1.4, 3.0, 7.0, and 11.7 T), showing a clear dependence of r_2_ on interparticle spacing, which decreased from 476 to 74 mM^−1^s^−1^ at B_0_ = 3.0 T as spacing increased. A plateau in r_2_ was observed under conditions of strong dipolar coupling, with maximum values approaching the asymptotic limit as E_D_ tended to infinity: r_2,max ED_ = 447 mM^−1^s^−1^ (B_0_ = 1.4 T), 471 mM^−1^s^−1^ (B_0_ = 3.0 T), 485 mM^−1^s^−1^ (B_0_ = 7.0 T), and 464 mM^−1^s^−1^ (B_0_ = 11.7 T). An interaction‐free r_2_ value was also determined, revealing the intrinsic effects of dipolar interactions and enabling demonstration of the influence of B_0_ on T_2_ CAs: r_2,min ED_ = 62 mM^−1^s^−1^ (B_0_ = 1.4 T), 74 mM^−1^s^−1^ (B_0_ = 3.0 T), 74 mM^−1^s^−1^ (B_0_ = 7.0 T), and 84 mM^−1^s^−1^ (B_0_ = 11.7 T). These results confirm a positive correlation between increasing field strength and r_2_, even in UHF‐MRI environments. Overall, this analysis enabled the identification of the asymptotic behavior of r_2_ under strong dipolar coupling and established a quantitative relationship between d_ij_, E_D,_ and r_2_, providing a solid framework for the rational design of iron oxide‐based T_2_ CAs.

A widely employed strategy in theranostic T_2_ CA design is the formation of core@shell nanoparticles, with a magnetic core and an inorganic shell (typically SiO_2_). This architecture enhances biocompatibility, enables surface functionalization, and facilitates controlled drug delivery.^[^ ^55^ ^]^ Our results indicate that shell thickness is a critical design parameter. Thicknesses below 1.4 nm generate very high E_D_ (>0.8 ·10^20^ J) and a plateau in r_2_, whereas layers within the range of 1.4–2.5 nm yield interparticle distances (11.7–14.0 nm), moderate E_D_ (0.46–0.89·10^20^ J), and r_2_ values approaching ≈90 % of the maximum. Thicker layers (≈4.0–4.8 nm) reduce r_2_ to ≈50 % due increased interparticle distances (17.0–18.6 nm) and consequently weaker dipolar interactions (E_D_ = 0.10–0.18·10^20^ J). Although E_D_ varies slightly with the applied magnetic field (B_0_ = 1.4–11.7 T), the optimal layer thickness range remains relatively constant. These findings provide concrete design parameters for iron oxide‐based T_2_ CAs, showing how shell engineering can maximize r_2_ while preserving surface functionalization and functionality.

Complementarily, the encapsulation of iron oxide‐based T_2_ CAs within larger spherical nanocarriers (typical of lipid‐based matrices, polymers, inorganic shells) represents another widely used strategy in theranostic nanomaterials, enabling combined imaging and therapeutic delivery.^[^ ^56^, ^57^, ^58^, ^59^, ^60^, ^61^ ^]^ However, the number of encapsulated MNPs directly determines the average d_ij_, and consequently, dipolar interactions and r_2._^[^ ^37^, ^62^ ^]^ Based on our model system, it was possible to estimate the optimal number of iron oxide MNPs per nanocarrier, considering homogeneous distribution of nanoparticles in the matrix volume and that each nanoparticle occupies a volume defined by its diameter plus the target d_ij_.

Assuming a typical 200‐nm nanocarrier, it is possible to anticipate the number of iron oxide nanoparticles that should be incorporated into the nanocomposite to achieve different r_2_ regimes. To reach the upper limit (r_2_ > 90% of r_2,max_), the interparticle distance should be d_ij_ < 12 nm, corresponding to more than 450 particles per nanocarrier. In the intermediate range (50–90% of r_2,max_), d_ij_ should be between 12 and 20 nm, with an estimated 170–450 particles per nanocarrier. Finally, the lower limit (r_2_<10% of r_2,max_) is reached when interparticle distances are large (d_ij_ > 30 nm), which corresponds to 70 particles or fewer per nanocarrier. Considering commonly used encapsulating materials such as lipids (ρ ≈ 0.9–1.0 g cm^−^^3^), polymers like PLGA (ρ ≈ 1.2 g cm^−^^3^), or inorganic SiO_2_ matrices (ρ ≈ 2.2 g cm^−^^3^), these particle estimations can be translated into corresponding iron oxide mass fractions (wt.%), a parameter widely used in nanocomposite design. To reach the upper limit of r_2_, more than ≈31 wt.% in lipids, ≈21 wt.% in PLGA, and ≈14 wt.% in SiO_2_ would be required. To achieve an intermediate efficacy range (50–90% of r_2,max_), mass fractions of ≈15–31 wt.% in lipids, ≈10–21 wt.% in PLGA, and ≈7–14 wt.% in SiO_2_ are recommended for nanocomposite design. Very low loadings (<4 wt.%, d_ij_≈ 30 nm) lead to a near‐complete loss of the r_2_ contribution induced by E_D_. In summary, these results provide a quantitative framework linking dipolar interactions to r_2_, from which optimal ranges can be calculated as a function of shell thickness and interparticle spacing. Such parameters can be extrapolated to other nanocomposite systems and systematically tuned to guide the rational design of iron oxide‐based T_2_ CAs.

## 4 Experimental Section

### Synthesis of Iron Oxide in Organic Media (IO@OA)

The synthesis of MNPs with a size of 9.0 ± 0.6 nm was carried out following the thermal decomposition method previously described elsewhere with slight modifications.^[^ ^63^, ^64^ ^]^ First, iron acetylacetonate (12 mmol), 1,2‐hexadecanediol (48 mmol), oleic acid (96 mmol) and oleylamine (24 mmol) were dissolved in benzyl ether (120 mL) at 110 °C for 60 min with mechanical stirring at 400 rpm under vacuum. Then, under a flow of nitrogen and reflux, the temperature was increased following a ramp of 8 °C min^−1^ to a temperature of 210 °C, remaining 2 h at this temperature. Then, the temperature was increased up to 300 °C following a heating ramp of 5 °C min^−1^, maintained for 1 h. Finally, the reaction was allowed to cool down to room temperature. Once the solution was cooled, the MNPs were precipitated by the addition of 50 mL of ethanol and centrifuged (4000 rpm, 10 min). The black precipitate was resuspended in a mixture of 50 mL of toluene and 500 µL of OA and OAm. Subsequently, the solution was centrifuged, the precipitate was discarded in order to eliminate possible aggregates, and the supernatant was recovered and redispersed in 50 mL of ethanol. After a new centrifugation cycle, the precipitate was resuspended in organic medium (cyclohexane).

### Transfer to Aqueous Media (IO@PAA)

MNPs dispersed in organic medium (Fe_3_O_4_@OA) were transfer to aqueous media by a ligand exchange process following previously reported work.^[^ ^65^ ^]^ 1 mL of Fe_3_O_4_@OA MNPs dispersed in CHX were redispersed in a solution of 11.32 mg of PAA in 50 mL of DMSO. The amount of PAA employed was adjusted following a molar ratio of the exchange ligand‐to‐NP surface Fe atoms at 5:1. The mixture was kept under magnetic stirring for 48 h at 700 rpm. Finally, the MNPs were precipitated by centrifugation (14 000 rpm, 30 min) and redispersed in H_2_O.

### Coating with Non‐Magnetic SiO_2_ Spacers (IO@SiO_2_)

MNPs dispersed in organic medium (IO@OA) were coated with SiO_2_ shells ranging from δ  = 2.1 nm to δ  = 9.9 nm thickness. The synthesis was carried out through the microemulsion method, previously described in other works.^[^ ^40^ ^]^ Initially, the corresponding amount of IGEPAL‐520 (Table S1, Supporting Information), was mixed with 231 mL of CHX under mechanical stirring at 350 rpm for 30 min. Subsequently, 34 mg of IO@OA MNPs dispersed in CHX were poured into the mixture and stirred mechanically at 350 rpm for another 30 min. NH_4_OH and TEOS were then added in the appropriate proportions (Table S1, Supporting Information), and the mixture was subjected to mechanical stirring at 350 rpm for 16 h while being shielded from solar radiation and light sources. Then, the corresponding amount of 2‐propanol was added until a flocculation of the MNPs was observed and the MNPs were retained using a permanent magnet, discarding the supernatant. The same procedure was repeated consecutively using a 1:1 mixture of 2‐propanol‐ethanol, ethanol and a 1:1 mixture of ethanol:H_2_O. Finally, the MNPs were washed twice with H_2_O, centrifuged at 9000 rpm for 30 min, and redispersed in H_2_O.

### Physicochemical and Magnetic Characterization of Nanoscale‐Distance‐Tuned MNPs

The characterization of the crystalline phases of the MNPs was performed by XRD on powder samples with a Philips PW1710 diffractometer (Cu K_a_ radiation source, λ = 1.54186 Å). Measurements were collected in the 2θ angle range between 10.00 and 80.00° with steps of 0.02 and 10 s per step. Morphology of the MNPs was characterized by TEM micrographs using a JEOL JEM‐1011 microscope (100 kV). The samples were prepared by dropping 7 µL of MNPs diluted in the media onto a carbon‐coated Cu grid followed by evaporation of the solvent at room temperature. The iron (Fe) concentration of the samples was determined by inductively coupled plasma emission spectroscopy (ICP‐OES, ICPE‐9000 Multitype ICP Emission Spectrometer, Shimadzu). Samples were prepared by digesting ≈10−20 µL of MNPs in 1 mL of HCl (37% v/v) overnight and diluting them with Milli‐Q water (10 mL total volume). The composition of the samples was analyzed using a TGA Perkin Elmer model 7 (Perkin Elmer, Waltham, MA, USA). Fourier transform infrared (FTIR) spectra of the samples were recorded with a Thermo Nicolet Nexus spectrometer (Thermo Fisher Scientific, Madrid, Spain) using the attenuated total reflectance (ATR) method from 4000 to 400 cm^−1^. DC magnetization curves of MNPs were measured using a Quantum Design Superconducting Quantum Interference Device (SQUID) magnetometer (Quantum Design, Darmstadt, Germany) in two different states. The powdered MNPs in an amount of ≈1 mg were placed in gelatin capsules. For the measurements of MNPs dispersed in H_2_O, 15 µL of the MNPs at the corresponding concentration were deposited in a polypropylene mold and sealed with GE varnish for cryogenic conditions. Subsequently, the mold was coated with cotton and Teflon and introduced into the sample holder held at both ends by two custom‐printed PLA stoppers obtained by 3D printing in order to fix the liquid sample in a centered position. ZFC and FC curves were taken at an applied magnetic field of 100 Oe from 10 to 350 K. The hysteresis loops were measured with an applied magnetic field between −25 and 25 kOe at 300 K. The dipolar interaction effects by DC magnetic measurements were obtained from the Henkel plots (δM) which are obtained by the ratio of the isothermal remanent magnetization (IRM, or M_R_(H)) and DC demagnetization (DCD, or M_D_(H)). AC magnetization curves were measured in a temperature range of 10–300 K with steps of 5 K, an excitation field of 2.5 Oe and driving frequencies varying from 50 to 750 Hz. More details on obtaining dipolar interactions in the Supporting Information.

### MRI Relaxation Properties of Nanoscale‐Distance‐Tuned Model System

Relaxation times were measured using a Minispec benchtop relaxometer (mq 60, Bruker, B_0_ = 1.41 T) operating at 60 MHz. Concentrations between 0 and 0.8 mm were used, and measurements were performed at a temperature of 37 °C. Samples with Fe concentrations between 0.0 and 0.8 mm were preheated to 37 °C and maintained at this temperature during the experiments. The relaxation times T_1_ (s) and T_2_ (s) were measured using standard saturation recovery (SR) and Carr‐Purcell‐ Meiboom‐Gill (CPMG) sequences, respectively, from which the longitudinal, r_1_ (mM^−1^s^−1^), and transverse, r_2_ (mM^−1^s^−1^), relaxivities were obtained. MR imaging was performed using a 3 T horizontal bore MR Solutions Benchtop scanner equipped with 48 G·cm^−1^ actively shielded gradients. To image the MNPs, a 56 mm diameter quadrature bird‐cage coil was used in transmit/receive mode. For the MRI phantom measurements, different Fe concentrations of MNPs in H_2_O were used from 0 to 80 µm. ≈100 µL of each MNP was placed on a custom‐printed PLA well plate, which was then placed at the center of the coil. T_2_‐weighted images were acquired using the fast spin echo (FSE) sequence with the following parameters: TE = 11 ms, TR = 12,000 ms, NA = 32. MRI images of phantoms were acquired with an image matrix of 256 × 252, FOV of 60 × 60 mm, six slices with a slice thickness of 0.5 mm, and a slice gap of 0 mm. Additionally, MR imaging was performed on a 7‐T Bruker Biospec 70/30 USR MRI system (Bruker Biospin GmbH, Ettlingen, Germany), interfaced to an AVANCE III console.  The BGA12 imaging gradient (maximum gradient strength 400 mT m^−1^) and a 40 mm diameter quadrature volume resonator was used for the acquisition.    For T_2_ maps imaging of the phantoms the following parameters were adopted: Bruker's MSME (Multi slice Spin echo) sequence was used. The TE values were varied in 128 steps ranging from 5.5 ms to 704 ms and TR 10 000 ms. All data were acquired with 2 averages, 160 ×1 60 points and a Field of View of 3.8 cm × 3.8 cm, 1 slice with a slice thickness of 1.5 mm. On the other hand, MR imaging was performed on a 11.7‐T Bruker Biospec 117/16USR MRI system (Bruker Biospin GmbH, Ettlingen, Germany), interfaced to an AVANCE III console, utilizing a 40 mm diameter quadrature volume resonator for acquisition. The same parameters as above were adopted for T_2_ maps imaging of the phantoms. Image analysis was performed using ImageJ software.

### Statistical Analysis

Statistical analyses were conducted using OriginPro 2016 (OriginLab Corporation, Northampton, MA, USA). Particle size distributions derived from TEM images were evaluated with ImageJ and reported as mean ± standard deviation (SD). To assess the goodness of fit of the particle size data, the Distribution Fit tool in OriginPro was employed. Measurements of hydrodynamic diameter, ζ‐potential, iron content, relaxometric parameters, and MRI analysis were performed in triplicate (n = 3) and expressed as mean ± SD.

## Conflict of Interest

The authors declare no conflict of interest.

## Supporting information



Supporting Information

## Data Availability

The data that support the findings of this study are available from the corresponding author upon reasonable request.

## References

[1] Z. Zhou , L. Yang , J. Gao , X. Chen , Adv. Mater. 2019, 31, 1804567. 10.1002/adma.201804567 PMC6392011 30600553

[2] M. Jeon , M. V. Halbert , Z. R. Stephen , M. Zhang , Adv. Mater. 2021, 33, 1906539. 10.1002/adma.201906539 PMC8022883 32495404

[3] E. Terreno , D. D. Castelli , A. Viale , S. Aime , Chem. Rev. 2010, 110, 3019. 20415475 10.1021/cr100025t

[4] J. Estelrich , M. J. Sánchez‐Martín , M. A. Busquets , Int. J. Nanomed. 2015, 10, 1727. 10.2147/IJN.S76501 PMC4358688 25834422

[5] J. V. Jokerst , S. S. Gambhir , Acc. Chem. Res. 2011, 44, 1050. 21919457 10.1021/ar200106e PMC3196845

[6] C. Catana , A. R. Guimaraes , B. R. Rosen , J. Nucl. Med. 2013, 54, 815. 23492887 10.2967/jnumed.112.112771 PMC3801211

[7] X. Liu , Z. Liang , H. Du , B. Zhang , Q. Wang , S. Xie , L. Xiao , Y. Chen , Y. Wang , F. Li , D. Ling , Nano Lett. 2024, 24, 6696. 38796774 10.1021/acs.nanolett.4c01389

[8] J. Wang , Y. Jia , Q. Wang , Z. Liang , G. Han , Z. Wang , J. Lee , M. Zhao , F. Li , R. Bai , D. Ling , Adv. Mater. 2021, 33, 2004917. 10.1002/adma.202004917 33263204

[9] Q. Cheng , Y. Chang , D. Zhang , X. Zhao , Z. Xiao , T. Chen , C. Shi , L. Luo , ACS Nano 2024, 18, 41. 10.1021/acsnano.4c00516 39370780

[10] Z. Liang , Q. Wang , H. Liao , M. Zhao , J. Lee , C. Yang , F. Li , D. Ling , Nat. Commun. 2021, 590, E12. 10.1038/s41467-021-24055-2 PMC8219830 34158498

[11] O. Kraff , A. Fischer , A. M. Nagel , C. Mönninghoff , M. E. Ladd , J. Magn. Reson. Imaging 2015, 41, 13. 24478137 10.1002/jmri.24573

[12] A. Banerjee , B. Blasiak , A. Dash , B. Tomanek , F. C. J. M. van Veggel , S. Trudel , Chem. Phys. Rev. 2022, 3, 011304.

[13] J. Dai , Z. Liu , L. Wang , G. Huang , S. Song , C. Chen , T. Wu , X. Xu , C. Hao , Y. Bian , E. A. Rozhkova , Z. Chen , H. Yang , J. Am. Chem. Soc. 2023, 145, 1108. 36622303 10.1021/jacs.2c10749

[14] D. Ni , W. Bu , E. B. Ehlerding , W. Cai , J. Shi , Chem. Soc. Rev. 2017, 46, 7438. 29071327 10.1039/c7cs00316a PMC5705441

[15] S. Song , Q. Wang , J. Xie , J. Dai , D. Ouyang , G. Huang , Y. Guo , C. Chen , M. Wu , T. Huang , J. Ruan , X. Cheng , X. Lin , Y. He , E. A. Rozhkova , Z. Chen , H. Yang , Adv. Healthcare Mater. 2023, 12, 2301437. 10.1002/adhm.202301437 37379009

[16] Y. Gan , J. Zhang , S. Lei , M. Yan , W. Xie , X. Qi , H. Wang , J. Xiao , S. Chen , S. Li , G. Tian , G. Zhang , Z. Wu , Chem. Eng. J. 2022, 429, 132255.

[17] C. Y. Chou , M. Abdesselem , C. Bouzigues , M. Chu , A. Guiga , T. H. Huang , F. Ferrage , T. Gacoin , A. Alexandrou , D. Sakellariou , Sci. Rep. 2017, 7, 44770. 28317892 10.1038/srep44770 PMC5357940

[18] S. Laurent , D. Forge , M. Port , A. Roch , C. Robic , L. Vander Elst , R. N. Muller , Chem. Rev. 2008, 108, 2064. 18543879 10.1021/cr068445e

[19] G. E. Hagberg , K. Scheffler , Contrast Media Mol. Imaging 2013, 8, 456. 24375901 10.1002/cmmi.1565

[20] L. Helm , Chimia (Aarau) 2011, 65, 696. 22026182 10.2533/chimia.2011.696

[21] J. Zeng , L. Huo , Z. Wang , X. Sun , Y. Guo , M. Li , M. Tan , S. Zhu , J. Fang , Z. Zhao , Chem. Mater. 2023, 35, 7643.

[22] S. Alzola‐Aldamizetxebarria , L. Fernández‐Méndez , D. Padro , J. Ruíz‐Cabello , P. Ramos‐Cabrer , ACS Omega 2022, 7, 36905. 36312407 10.1021/acsomega.2c03549 PMC9609087

[23] J. Mohapatra , S. Nigam , J. George , A. C. Arellano , P. Wang , J. P. Liu , Mater. Today Phys. 2023, 32, 101103. 10.1016/j.mtphys.2023.101003 PMC12308503 40740662

[24] G. Si , Y. Du , P. Tang , G. Ma , Z. Jia , X. Zhou , D. Mu , Y. Shen , Y. Lu , Y. Mao , C. Chen , Y. Li , N. Gu , Natl. Sci. Rev. 2024, 11, nwae057. 38577664 10.1093/nsr/nwae057 PMC10989670

[25] A. Banerjee , B. Blasiak , A. Dash , B. Tomanek , F. C. J. M. van Veggel , S. Trudel , Chem. Phys. Rev. 2022, 3, 011304.

[26] R. Weissleder , S. Saini , D. D. Stark , J. Wittenberg , J. T. Ferrucci , Am. J. Roentgenol. 1988, 150, 3. 3257610 10.2214/ajr.150.3.561

[27] Z. Zhou , R. Bai , J. Munasinghe , Z. Shen , L. Nie , X. Chen , ACS Nano 2017, 11, 5227. 28613821 10.1021/acsnano.7b03075 PMC9617470

[28] H. Du , Q. Wang , B. Zhang , Z. Liang , C. Huang , D. Shi , F. Li , D. Ling , Adv. Mater. 2024, 36, 2401538. 10.1002/adma.202401538 38738793

[29] J. S. Choi , S. Kim , D. Yoo , T. H. Shin , H. Kim , M. D. Gomes , S. H. Kim , A. Pines , J. Cheon , Nat. Mater. 2017, 16, 537. 28166216 10.1038/nmat4846

[30] H. Lu , A. Chen , X. Zhang , Z. Wei , R. Cao , Y. Zhu , J. Lu , Z. Wang , L. Tian , Nat. Commun. 2022, 50, 13. 10.1038/s41467-022-35655-x PMC9792454 36572677

[31] Z. Cai , C. Wu , L. Yang , D. Wang , H. Ai , ACS Biomater. Sci. Eng. 2020, 6, 2533. 33463262 10.1021/acsbiomaterials.9b01198

[32] Z. Zhou , R. Tian , Z. Wang , Z. Yang , Y. Liu , G. Liu , R. Wang , J. Gao , J. Song , L. Nie , X. Chen , Nat. Commun. 2017, 8, 15468. 28516947 10.1038/ncomms15468 PMC5454366

[33] W. Liu , S. Y. Yin , Y. Hu , T. Deng , J. Li , ACS Appl. Mater. Interfaces 2022, 14, 2629. 35000378 10.1021/acsami.1c22747

[34] C. Paquet , H. W. De Haan , D. M. Leek , H. Y. Lin , B. Xiang , G. Tian , A. Kell , B. Simard , ACS Nano 2011, 5, 3104. 21428441 10.1021/nn2002272

[35] V. Dahanayake , T. Lyons , B. Kerwin , O. Rodriguez , C. Albanese , E. Parasido , Y. Lee , E. Van Keuren , L. Li , E. Maxey , T. Paunesku , G. Woloschak , S. L. Stoll , ACS Appl. Mater. Interfaces 2021, 13, 39042. 34375073 10.1021/acsami.1c09232 PMC10506655

[36] Y. Liu , S. L. Ho , T. Tegafaw , D. Zhao , M. Y. Ahmad , A. K. Ali Al Saidi , H. Cha , S. Lee , H. Lee , S. Kim , M. Han , K. S. Chae , Y. Chang , G. H. Lee , Nanotechnology 2024, 35, 505101. 10.1088/1361-6528/ad8203 39353465

[37] S. Scialla , N. Genicio , B. Brito , M. Florek‐Wojciechowska , G. J. Stasiuk , D. Kruk , M. Bañobre‐López , J. Gallo , ACS Appl. Nano Mater. 2022, 5, 16462. 36569339 10.1021/acsanm.2c03537 PMC9778729

[38] I. Bok , B. Rauch , A. Ashtiani , A. Hai , Magn. Reson. Med. 2024, 91, 687. 37867452 10.1002/mrm.29898 PMC11489851

[39] C. Paquet , H. W. De Haan , D. M. Leek , H. Y. Lin , B. Xiang , G. Tian , A. Kell , B. Simard , ACS Nano 2011, 5, 3104. 21428441 10.1021/nn2002272

[40] P. García‐Acevedo , M. A. González‐Gómez , Á. Arnosa‐Prieto , L. de Castro‐Alves , Y. Piñeiro , J. Rivas , Adv. Sci. 2023, 10, 2203397. 10.1002/advs.202203397 PMC9929252 36509677

[41] V. S. Coker , A. M. T. Bell , C. I. Pearce , R. A. D. Patrick , G. van der Laan , J. R. Lloyd , Am. Mineral. 2008, 93, 540.

[42] A. Rajan , M. Sharma , N. K. Sahu , Sci. Rep. 2020, 10, 15045. 32963264 10.1038/s41598-020-71703-6 PMC7508873

[43] Y. A. Urian , J. J. Atoche‐Medrano , L. T. Quispe , L. León Félix , J. A. H. Coaquira , J. Magn. Magn. Mater. 2021, 525, 167686.

[44] L. M. Sanchez , D. A. Martin , V. A. Alvarez , J. S. Gonzalez , Colloids Surf., A 2018, 543, 28.

[45] S. L. C. Pinho , G. A. Pereira , P. Voisin , J. Kassem , V. Bouchaud , L. Etienne , J. A. Peters , L. Carlos , S. Mornet , C. F. G. C. Geraldes , J. Rocha , M. H. Delville , ACS Nano 2010, 4, 5339. 20795638 10.1021/nn101129r

[46] S. L. C. Pinho , S. Laurent , J. Rocha , A. Roch , M. H. Delville , S. Mornet , L. D. Carlos , L. Vander Elst , R. N. Muller , C. F. G. C. Geraldes , J. Phys. Chem. C 2012, 116, 2285.

[47] L. E. W. LaConte , N. Nitin , O. Zurkiya , D. Caruntu , C. J. O'Connor , X. Hu , G. Bao , J. Magn. Reson. Imaging 2007, 26, 1634. 17968941 10.1002/jmri.21194

[48] J. Gallo , B. I. Harriss , J. Hernández‐Gil , M. Bañobre‐López , N. J. Long , Nanoscale 2017, 9, 11318. 28762407 10.1039/c7nr01733b

[49] M. E. Ladd , P. Bachert , M. Meyerspeer , E. Moser , A. M. Nagel , D. G. Norris , S. Schmitter , O. Speck , S. Straub , M. Zaiss , Prog. Nucl. Magn. Reson. Spectrosc. 2018, 109, 1. 30527132 10.1016/j.pnmrs.2018.06.001

[50] H. Du , Q. Wang , Z. Liang , Q. Li , F. Li , D. Ling , Nanoscale 2022, 14, 17483. 36413075 10.1039/d2nr04979a

[51] F. Brero , P. Arosio , M. Albino , D. Cicolari , M. Porru , M. Basini , M. Mariani , C. Innocenti , C. Sangregorio , F. Orsini , A. Lascialfari , Nanomaterials 2023, 13, 804. 36903682 10.3390/nano13050804 PMC10005490

[52] M. Cho , J. Villanova , D. M. Ines , J. Chen , S. S. Lee , Z. Xiao , X. Guo , J. A. Dunn , D. D. Stueber , P. Decuzzi , V. L. Colvin , J. Phys. Chem. C 2023, 127, 1057.

[53] N. Lee , T. Hyeon , Chem. Soc. Rev. 2012, 41, 2575. 22138852 10.1039/c1cs15248c

[54] J. García‐Otero , M. Porto , J. Rivas , A. Bunde , Phys. Rev. Lett. 2000, 64, 167. 10.1103/PhysRevLett.84.167 11015861

[55] Y. Zou , Z. Sun , Q. Wang , Y. Ju , N. Sun , Q. Yue , Y. Deng , S. Liu , S. Yang , Z. Wang , F. Li , Y. Hou , C. Deng , D. Ling , Y. Deng , Chem. Rev. 2025, 125, 972. 39729245 10.1021/acs.chemrev.4c00710

[56] B. Rezaei , P. Yari , S. M. Sanders , H. Wang , V. K. Chugh , S. Liang , S. Mostufa , K. Xu , J. P. Wang , J. Gómez‐Pastora , K. Wu , Small 2024, 20, 2304848. 10.1002/smll.202304848 37732364

[57] N. Lee , D. Yoo , D. Ling , M. H. Cho , T. Hyeon , J. Cheon , Chem. Rev. 2015, 115, 10637. 26250431 10.1021/acs.chemrev.5b00112

[58] J. V. R. Rocha , R. F. Krause , C. E. Ribeiro , N. C. de. A. Oliveira , L. Ribeiro de Sousa , J. Leandro Santos , S. de. M. Castro , M. C. Valadares , M. Cunha Xavier Pinto , M. V. Pavam , E. M. Lima , S. Antônio Mendanha , A. F. Bakuzis , ACS Appl. Mater. Interfaces 2025, 17, 13094. 38973727 10.1021/acsami.4c03434 PMC11891835

[59] S. Lee , J. Park , H. N. Jung , S. Li , Z. Lin , H. J. Im , Adv. Healthcare Mater. 2025.

[60] C. V. Rocha , A. P. Magalhães , V. Gonçalves , L. Diego‐González , M. Bañobre‐López , J. Gallo , J. Mater. Chem. B 2025, 13, 5808. 40265214 10.1039/d5tb00148j

[61] Z. Cai , C. Wu , L. Yang , D. Wang , H. Ai , ACS Biomater. Sci. Eng. 2020, 6, 2533. 33463262 10.1021/acsbiomaterials.9b01198

[62] R. Grillo , J. Gallo , D. G. Stroppa , E. Carbó‐Argibay , R. Lima , L. F. Fraceto , M. Bañobre‐López , ACS Appl. Mater. Interfaces 2016, 8, 25777. 27595772 10.1021/acsami.6b08663

[63] A. Pardo , B. Pelaz , J. Gallo , M. Bañobre‐López , W. J. Parak , S. Barbosa , P. Del Pino , P. Taboada , Chem. Mater. 2020, 32, 2220.

[64] J. Meng , P. Zhang , Q. Chen , Z. Wang , Y. Gu , J. Ma , W. Li , C. Yang , Y. Qiao , Y. Hou , L. Jing , Y. Wang , Z. Gu , L. Zhu , H. Xu , X. Lu , M. Gao , Adv. Mater. 2022, 34. 10.1002/adma.202202168 35362203

[65] Y. Xu , Y. Qin , S. Palchoudhury , Y. Bao , Langmuir 2011, 27, 8990. 21644795 10.1021/la201652h

