# Supplementary material for: Unveiling Dipolar Interaction‐Driven Magnetic Field Inhomogeneities in T2 MRI Contrast Agents
Source: Adv Sci (Weinh). 2025 Nov 4;13(3):e10356. doi: 10.1002/advs.202510356 (PMC12806336; doi:10.1002/advs.202510356)
Supplement: Supplementary file 1 — Supporting Information [file ADVS-13-e10356-s001.docx]

Supporting Information

Unveiling Dipolar Interaction-Driven Magnetic Field Inhomogeneities in T_2_ MRI Contrast Agents

Pelayo García-Acevedo,* Yolanda Piñeiro, Juan Gallo, Pedro Ramos-Cabrer, Ramón Iglesias-Rey, José Rivas and Manuel Bañobre-López*

Pelayo García-Acevedo, Yolanda Piñeiro and José Rivas

NANOMAG Laboratory, Applied Physics Department, iMATUS Materials Institute and Health Research Institute of Santiago de Compostela (IDIS), Universidade de Santiago de Compostela, 15782, Santiago de Compostela, Spain

Pelayo García-Acevedo and Ramón Iglesias-Rey

Neuroimaging and Biotechnology Laboratory (NOBEL), Clinical Neurosciences Research Laboratory (LINC), Health Research Institute of Santiago de Compostela (IDIS), Santiago de Compostela, Spain

E-mail: pelayo.garcia.acevedo@sergas.es

Pedro Ramos-Cabrer

Center for Cooperative Research in Biomaterials (CIC biomaGUNE), Basque Research and Technology Alliance (BRTA), Donostia-San Sebastián, Spain.

Pelayo García-Acevedo, Juan Gallo and Manuel Bañobre-López

Advanced (magnetic) Theranostic Nanostructures Lab, International Iberian Nanotechnology Laboratory, Braga, Portugal

E-mail: pelayo.garcia@inl.int; manuel.banobre@inl.int

**Table S1.** Amount of NH_4_OH and TEOS to obtain different SiO_2_ layer thickness following the microemulsion method.

| Sample | IGEPAL-520  (g) | NH_4_OH  (mL) | TEOS  (mL) | SiO_2_ layer  (nm) |
| --- | --- | --- | --- | --- |
| IO@SiO_2_ – 2.1 nm | 5.0 | 0.7 | 0.8 | 2.15 |
| IO@SiO_2_ – 3.5 nm | 7.5 | 1.05 | 1.2 | 3.50 |
| IO@SiO_2_ – 5.3 nm | 10.0 | 1.4 | 1.6 | 5.30 |
| IO@SiO_2_ – 9.9 nm | 15.0 | 2.8 | 3.2 | 9.85 |


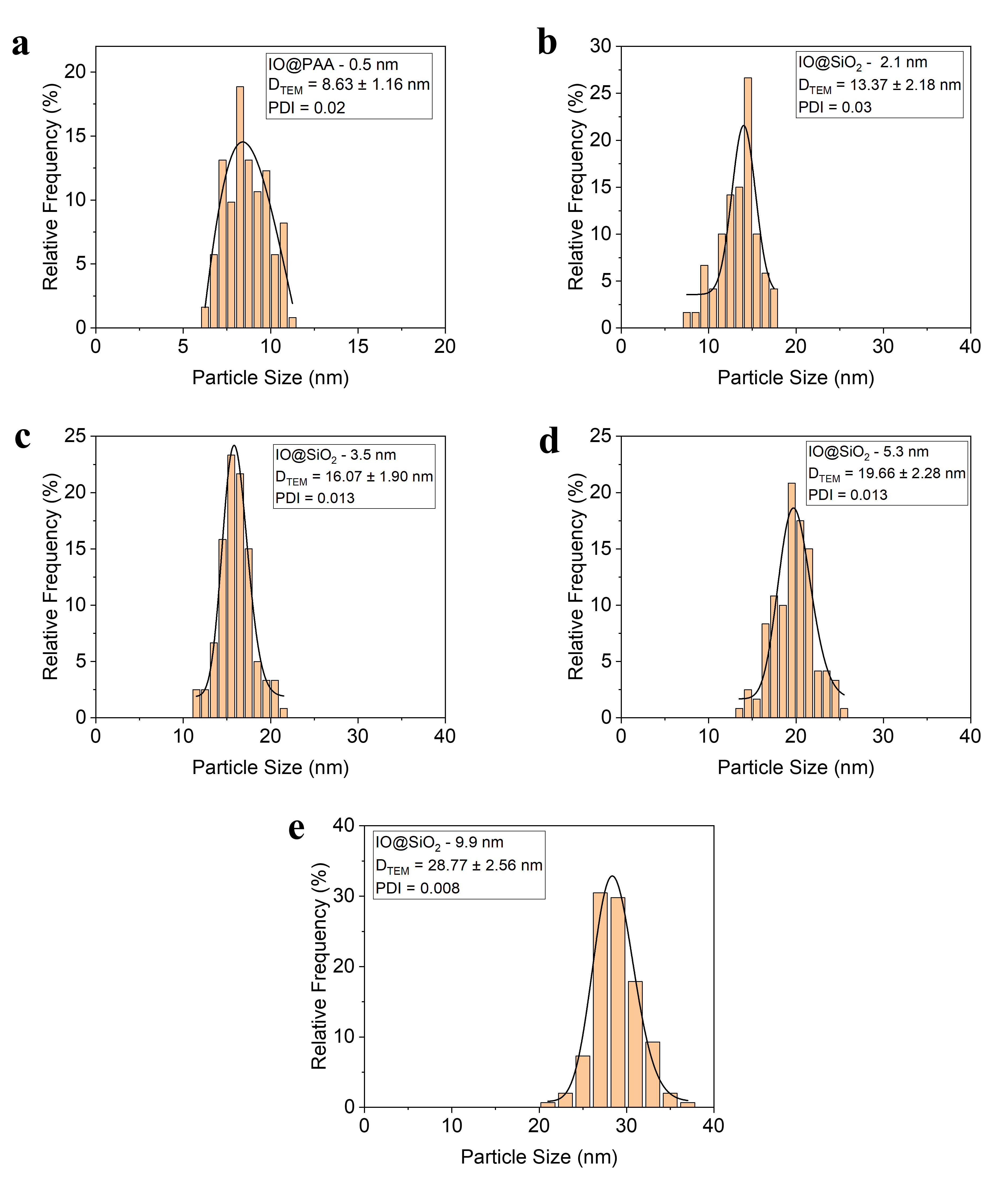


**Figure S1**. Size distribution histograms obtained by TEM micrographs of the IOMNP set: **(a)** IO@PAA - δ=0.5 nm, **(b)** IO@SiO_2_ - δ=2.1 nm, **(c)** IO@SiO_2_ - δ=3.5 nm, **(d)** IO@SiO_2_ - δ=5.3 nm, and **(e)** IO@SiO_2_ - δ=9.9 nm. Size distribution was performed using Image J software.


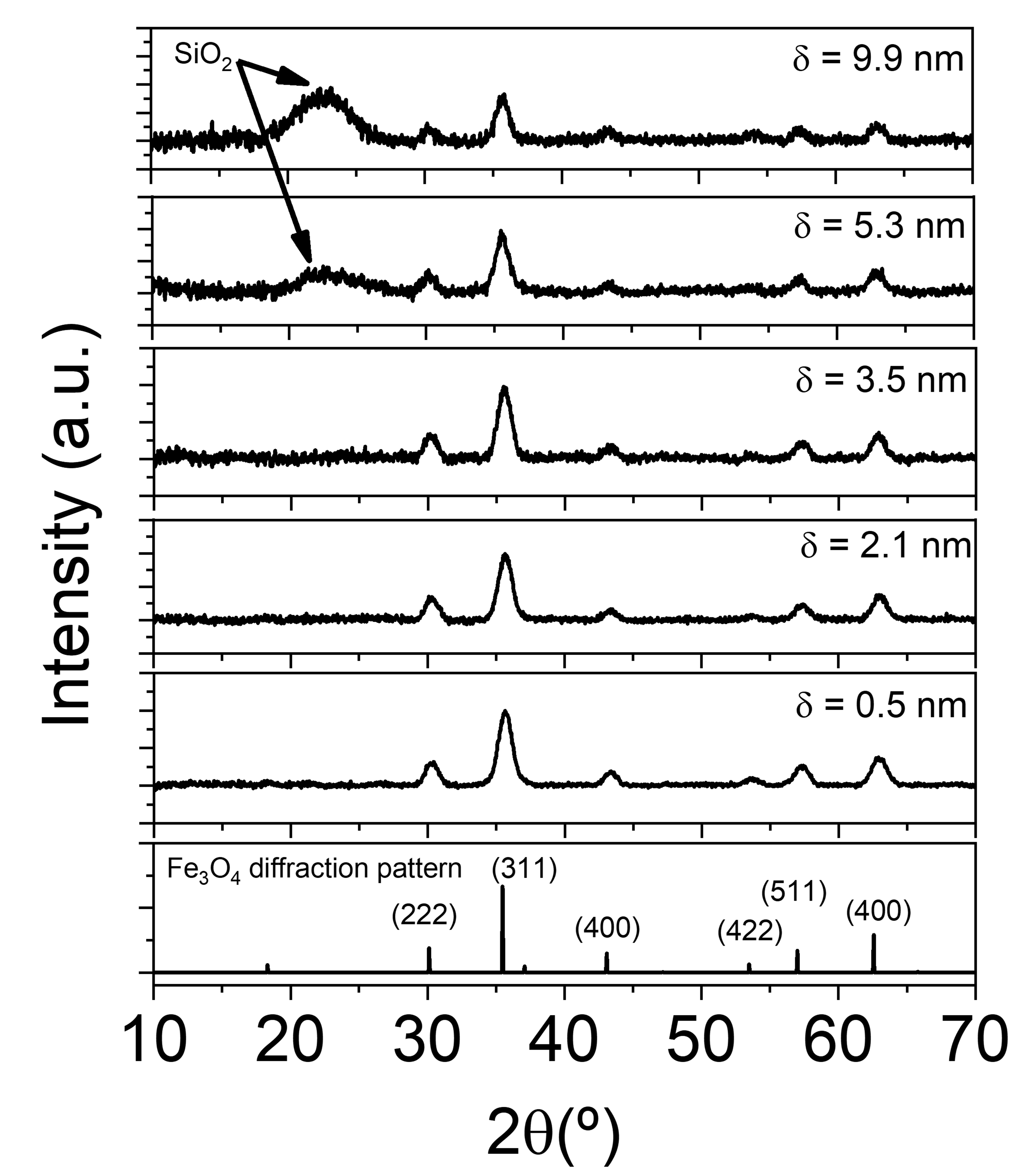


**Figure S2**. XRD patterns of, IO@PAA (δ = 0.5 nm), IO@SiO₂ (δ = 2.1 nm), IO@SiO₂ (δ = 3.5 nm), IO@SiO₂ (δ = 5.3 nm), and IO@SiO₂ (δ = 9.9 nm). The theoretical diffraction pattern of magnetite is shown at the bottom for reference.


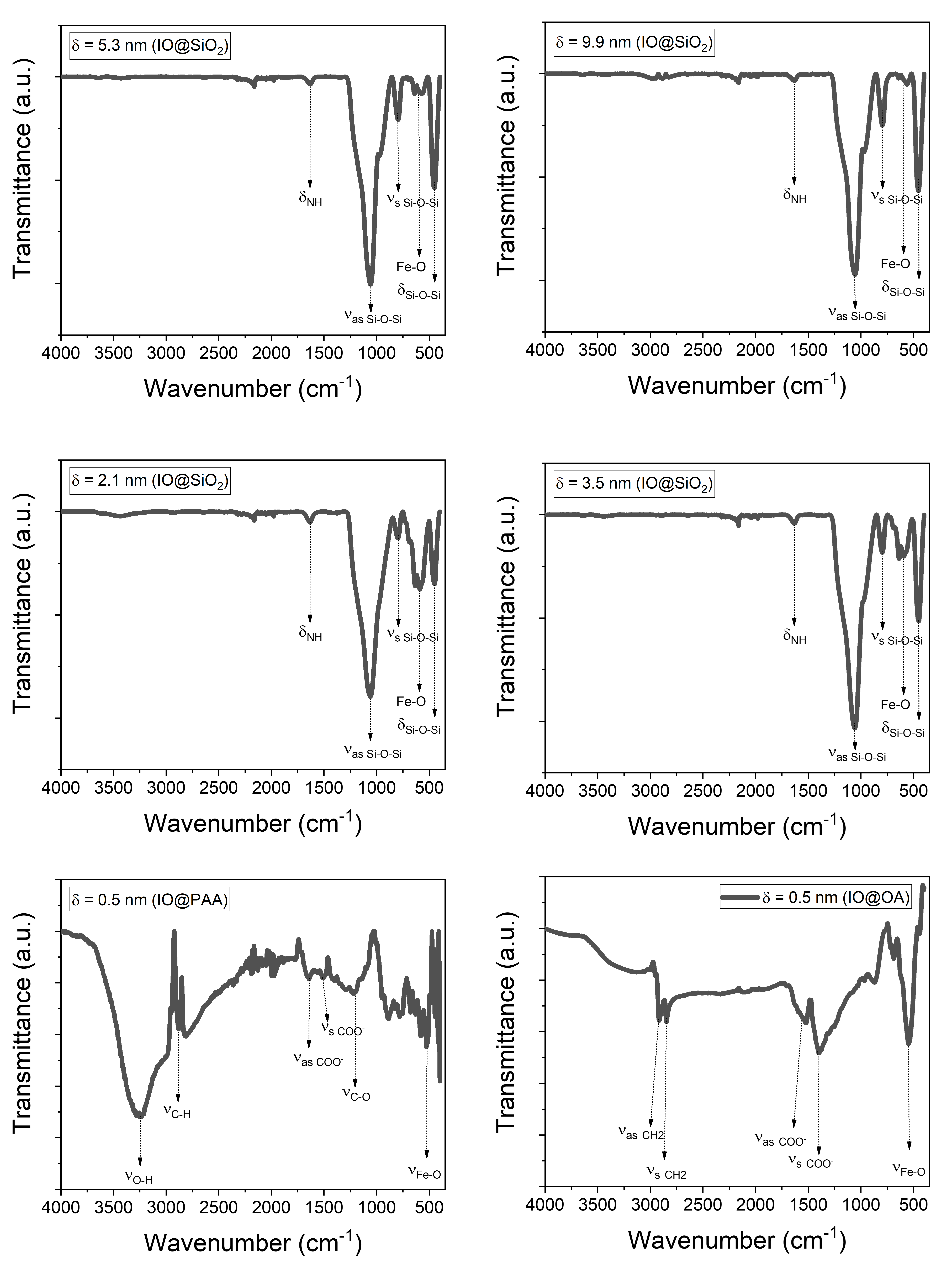


**Figure S3**. FT-IR spectra (4000–400 cm⁻¹) of IO@OA, IO@PAA (δ = 0.5 nm), IO@SiO₂ (δ = 2.1 nm), IO@SiO₂ (δ = 3.5 nm), IO@SiO₂ (δ = 5.3 nm), and IO@SiO₂ (δ = 9.9 nm).


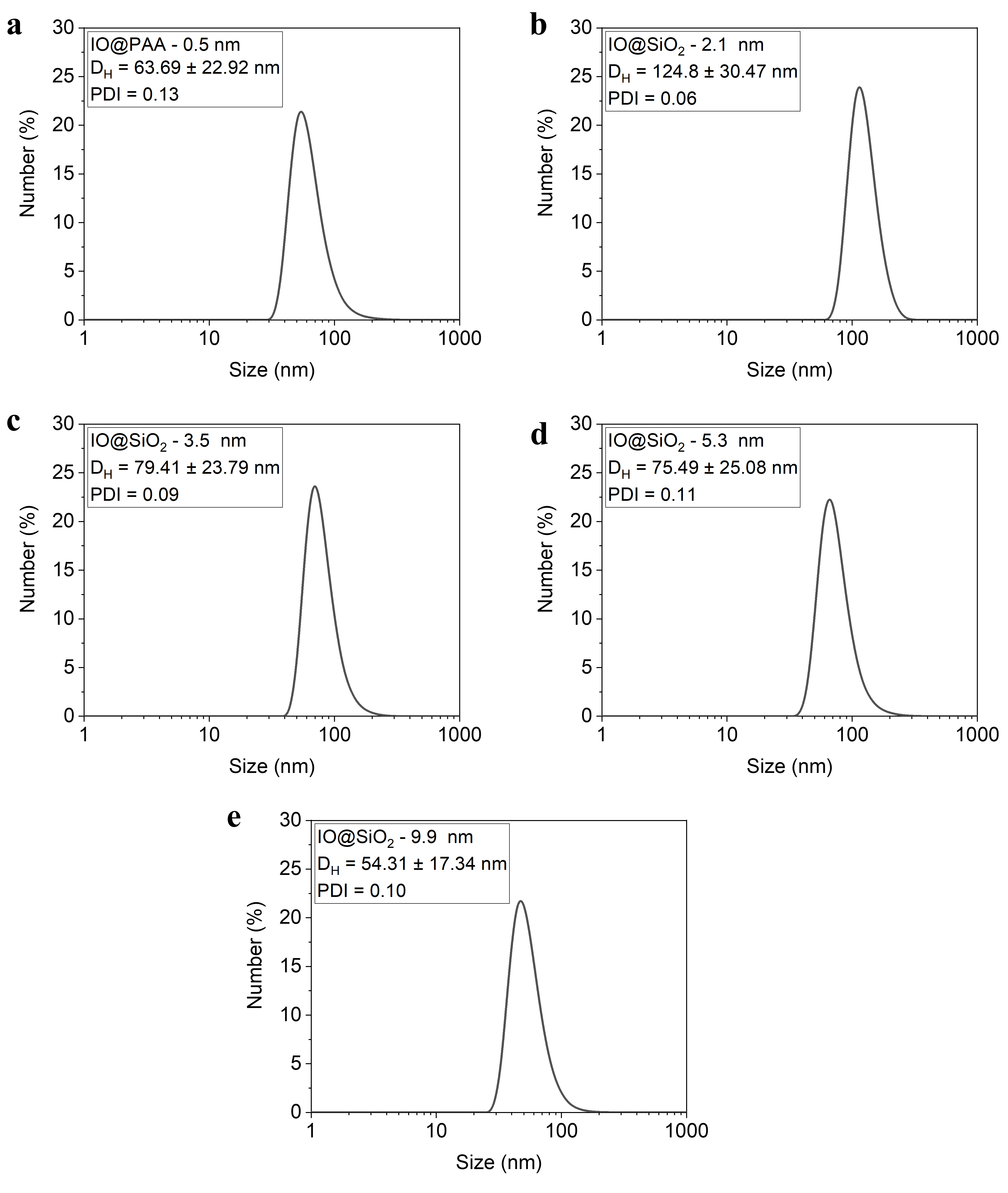


**Figure S4.** Hydrodynamic size distribution obtained by DLS measurements of **(a)** IO@PAA - δ=0.5 nm, **(b)** IO@SiO_2_ - δ=2.1 nm, **(c)** IO@SiO_2_ - δ=3.5 nm, **(d)** IO@SiO_2_ - δ=5.3 nm, and **(e)** IO@SiO_2_ - δ=9.9 nm.


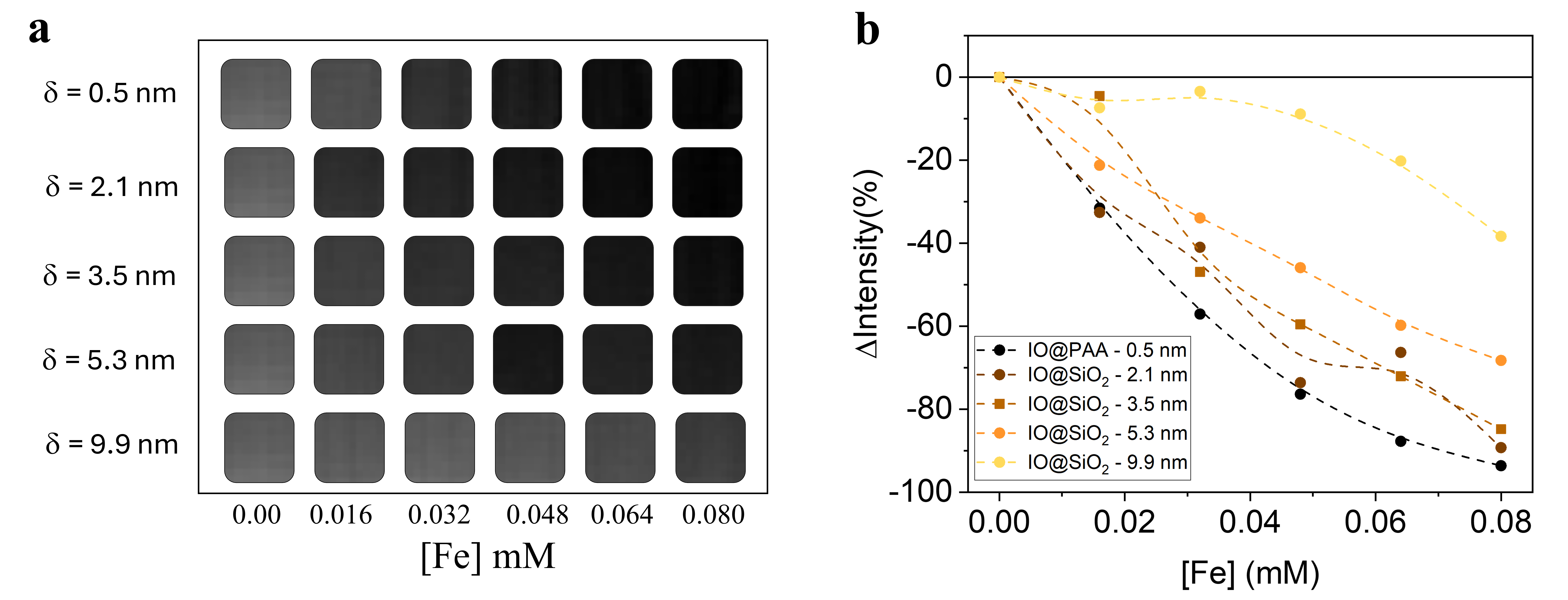


**Figure S5.** **(a)** T_2_ MRI signals employing a B_0_ = 3.0 T for the whole batch of MNPs, ranging from 0.5 nm spacer thickness (top) to 9.9 nm (bottom) using different concentrations (control, left) and 0.08 mM (right. **(b)** Variation of the signal intensity (ΔIntensity) normalized to the control signal (H_2_O) for MNPs with different silica shell thicknesses, obtained from the MRI T_2_ signals.


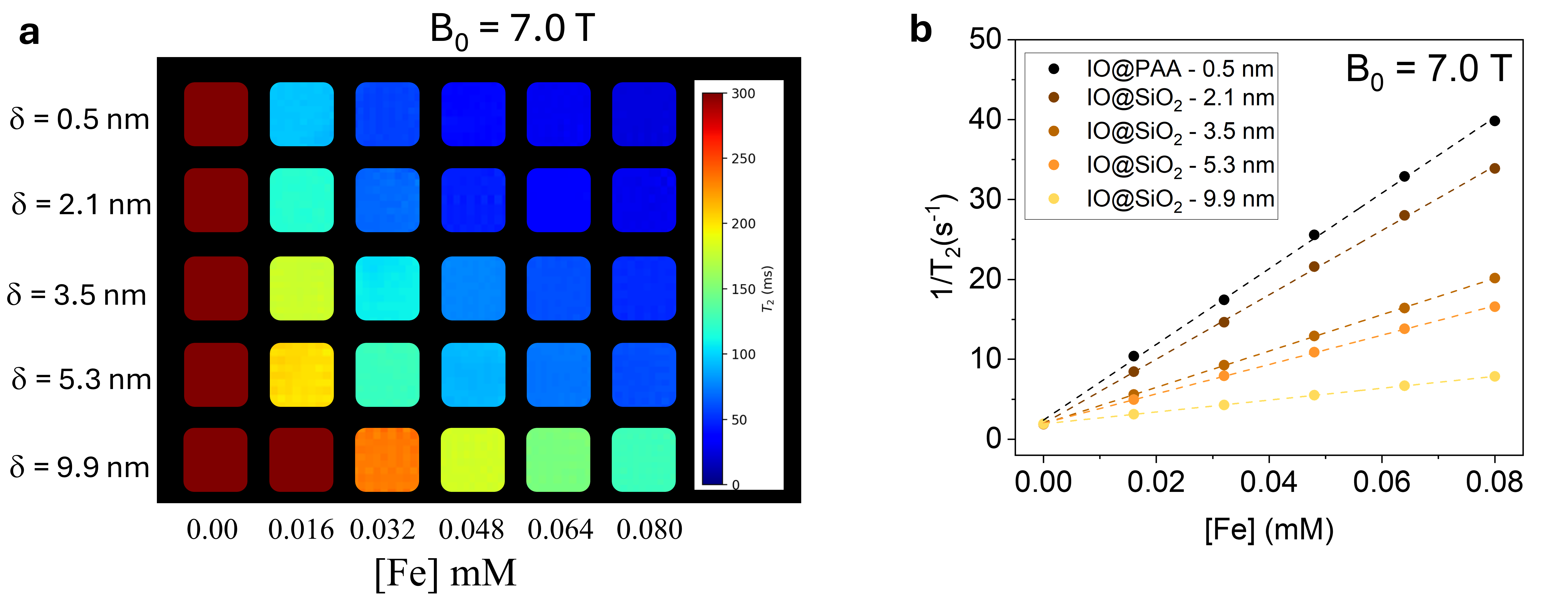


**Figure S6.** **(a)** T_2_ MRI maps employing a B_0_ = 7.0 T for the whole batch of MNPs, ranging from 0.5 nm spacer thickness (top) to 9.9 nm (bottom) using different concentrations (control, left) and 0.08 mM (right). **(b)** Transverse relaxation time, 1/T_2_ as a function of concentrations from 0.00 mM (control) to 0.8 mM for MNPs with different thicknesses, obtained from the MRI T_2_ maps.


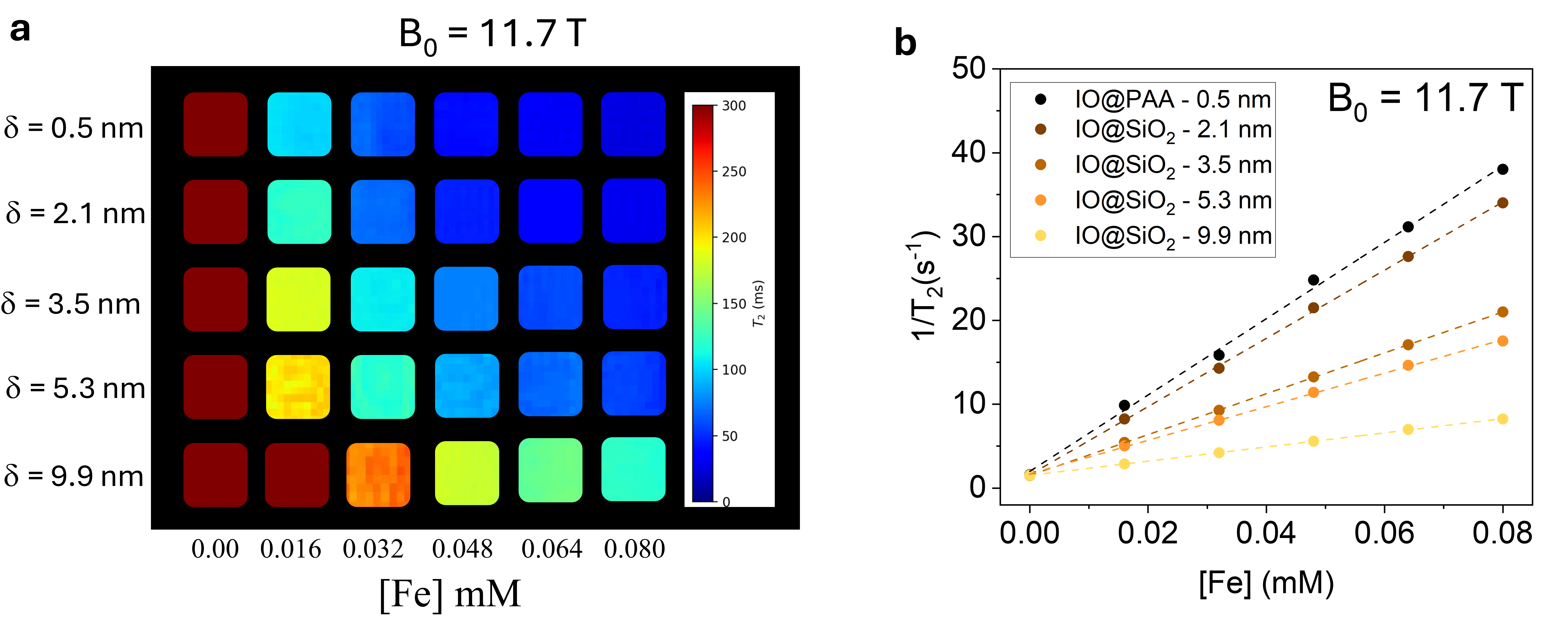


**Figure S7. (a)** T_2_ MRI maps employing a B_0_ = 11.7 T for the whole batch of MNPs, ranging from 0.5 nm spacer thickness (top) to 9.9 nm (bottom) using different concentrations (control, left) and 0. 8 mM (right). **(b)** Transverse relaxation time, 1/T_2_ as a function of concentrations from 0.00 mM (control) to 0.8 mM for MNPs with different thicknesses, from δ=0.5 nm (dark color) to δ =9.9 nm (yellow color), obtained from the MRI T_2_ maps.


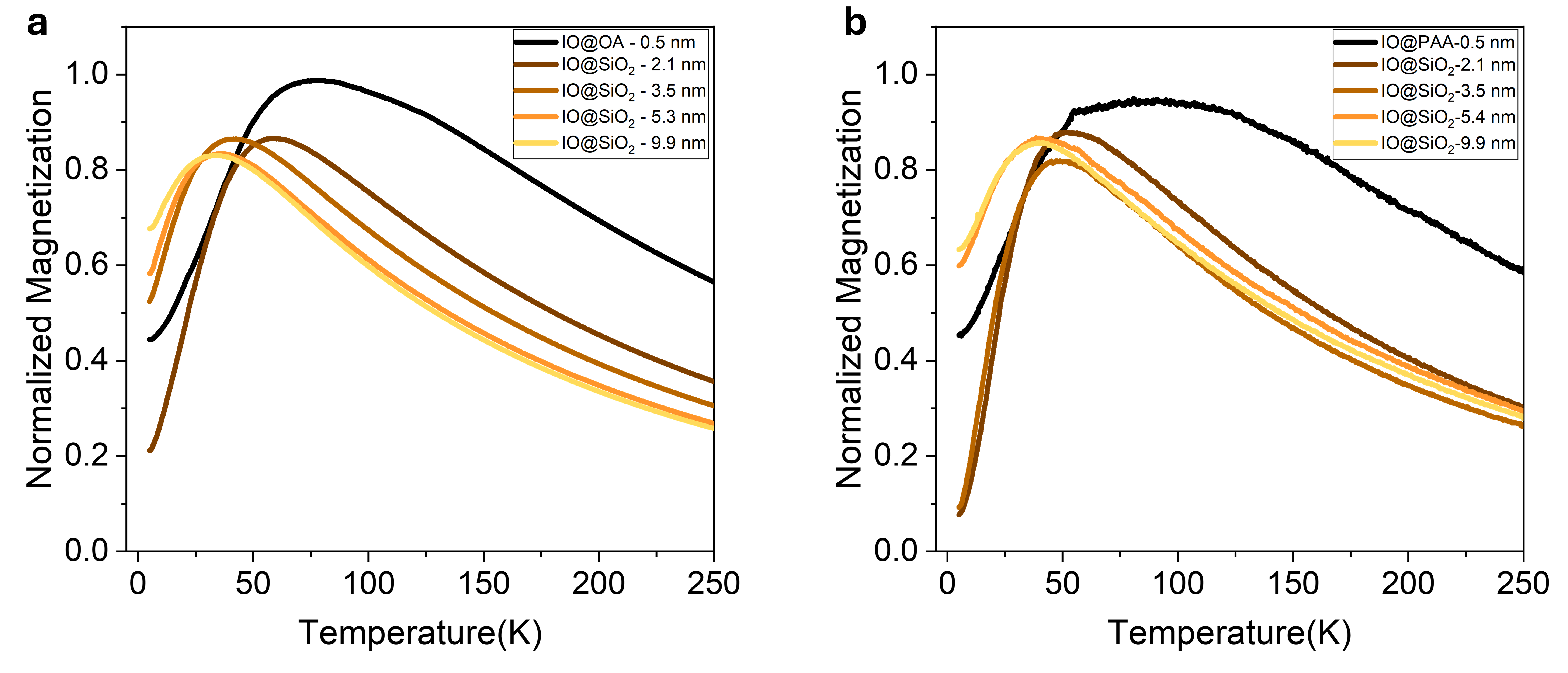


**Figure S8**. ZFC magnetization curves normalized to maximum magnetization of (a) powder and (b) aqueous-dispersed (0.8 mM) MNPs with different silica shell thicknesses, from δ=0.5 nm (dark color) to δ =9.9 nm (yellow color).


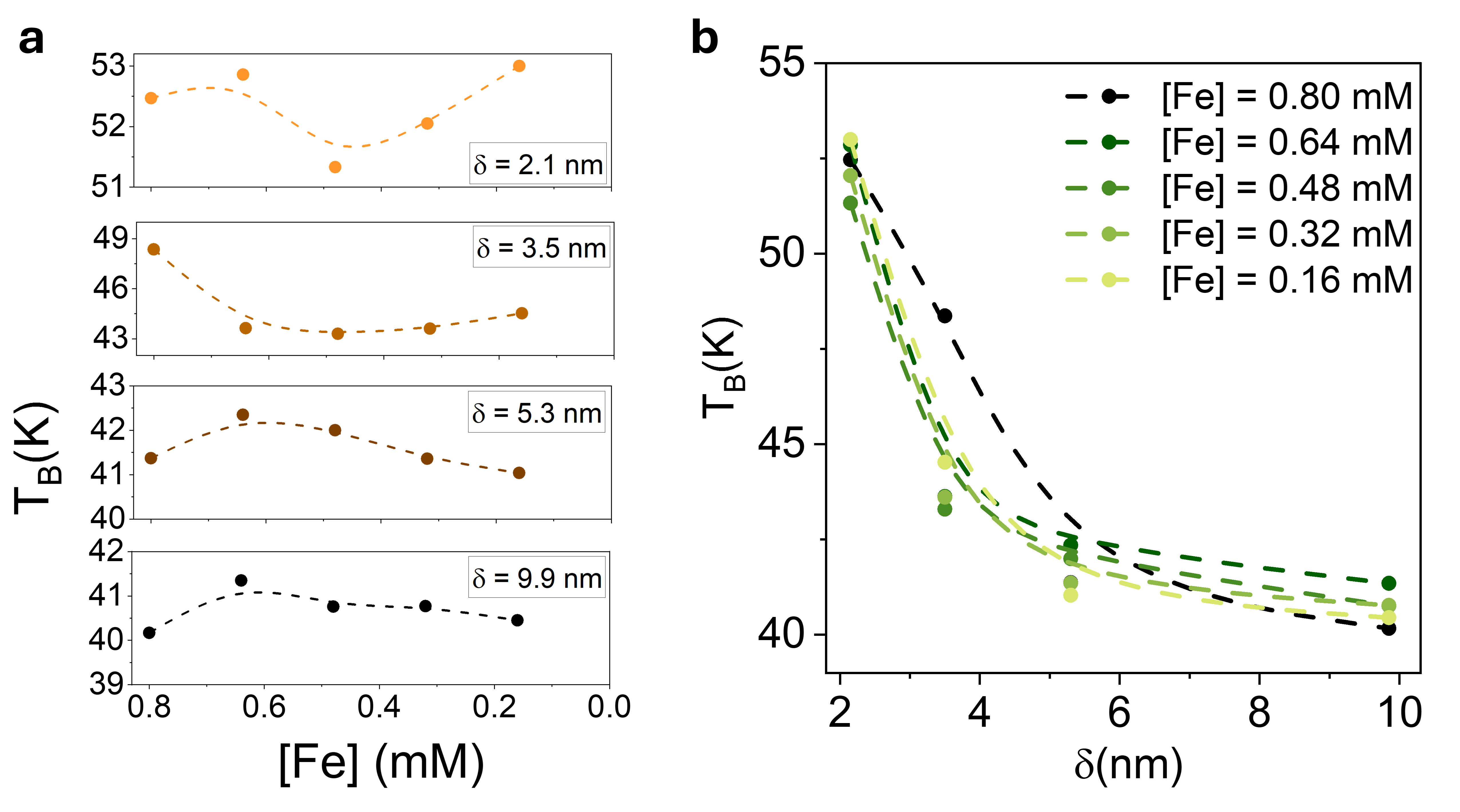


**Figure S9**. **(a)** Dependence of T_B_ with concentration for MNPs with different spacer thickness in the aqueous-dispersed state, from δ=2.1 nm (top, light tone) to δ=9.9 nm (bottom dark tone). **(b)** Dependence of T_B_ with spacer thickness, at different concentrations, from 0.80 mM (dark tone) to 0.16 mM (light tone).


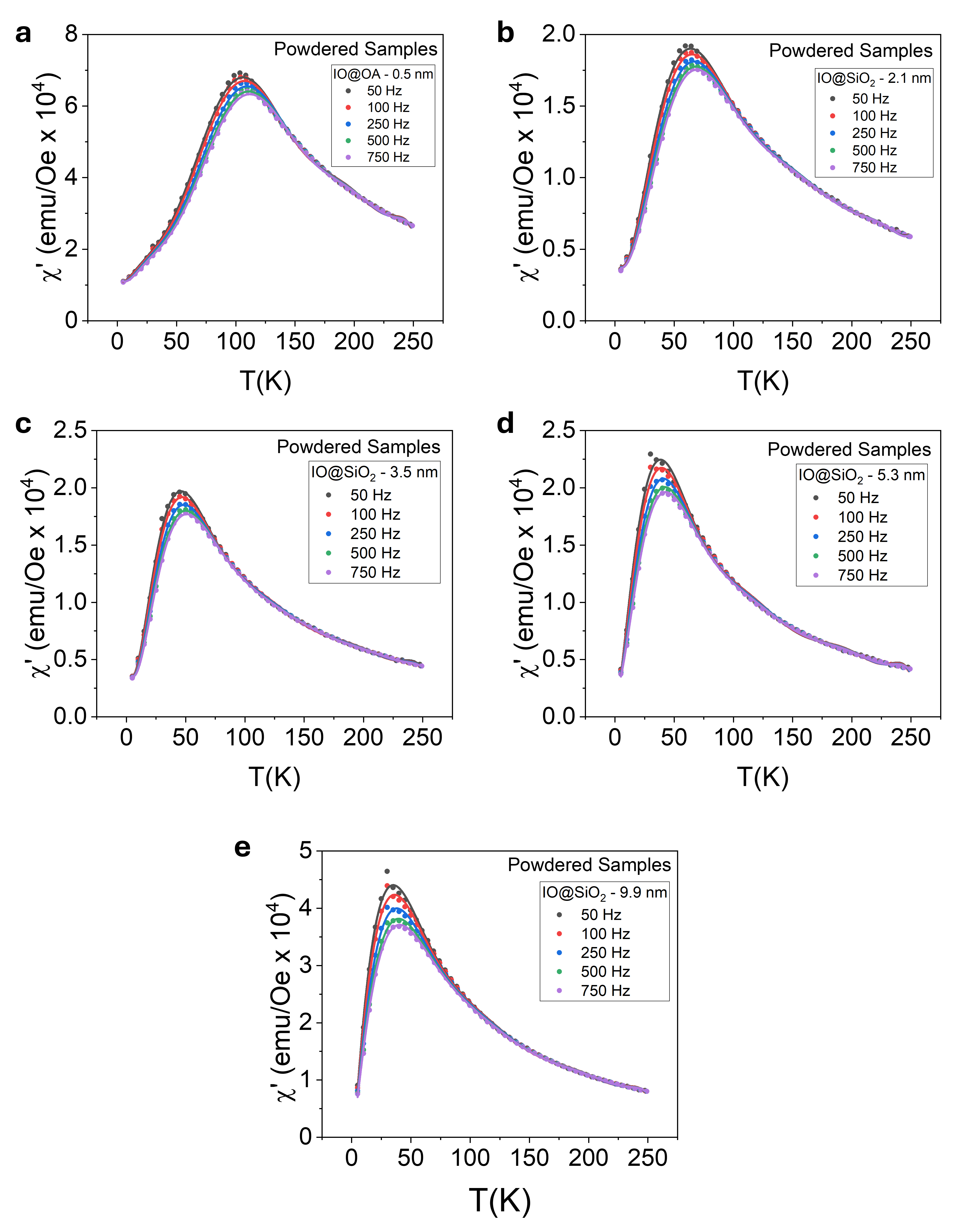


**Figure S10**. Temperature dependence of the real component χ’(T) for powdered MNPs with different spacer thickness: **(a)** IO@OA - δ=0.5 nm, **(b)** IO@SiO_2_ - δ=2.1 nm, **(c)** IO@SiO_2_ - δ=3.5 nm, **(d)** IO@SiO_2_ - δ=5.3 nm and (**e)** IO@SiO_2_ - δ=9.9 nm. The curves were obtained at the following frequencies: 50 Hz (gray), 100 Hz (red), 250 Hz (blue), 500 Hz (green), and 750 Hz (purple).


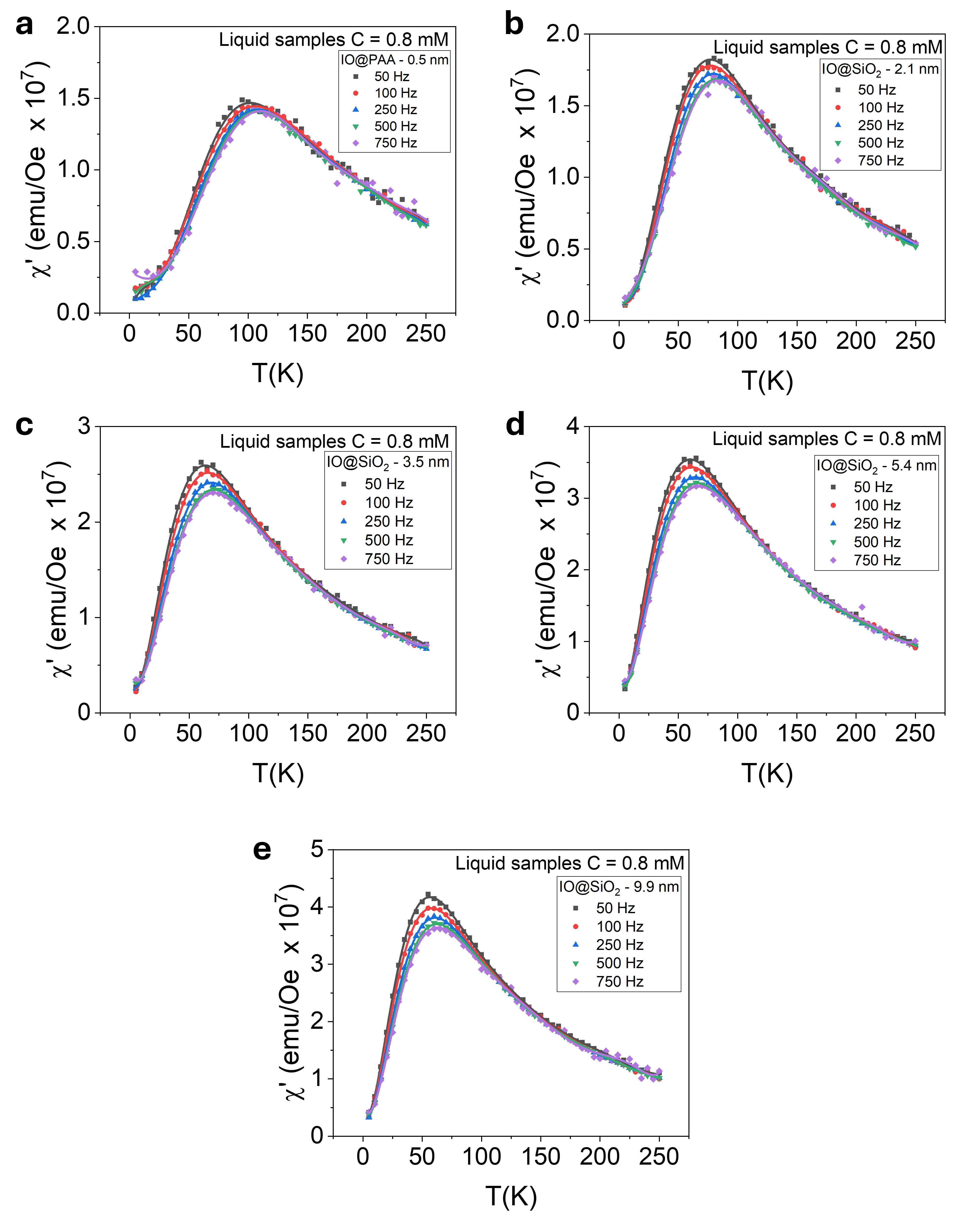


**Figure S11**. Temperature dependence of the real component χ’(T) for water-dispersed MNPs with different spacer thickness: **(a)** IO@PAA - δ=0.5 nm, **(b)** IO@SiO_2_ - δ=2.1 nm, **(c)** IO@SiO_2_ - δ=3.5 nm, **(d)** IO@SiO_2_ - δ=5.3 nm and **(e)** IO@SiO_2_ - δ=9.9 nm. The curves were obtained at the following frequencies: 50 Hz (gray), 100 Hz (red), 250 Hz (blue), 500 Hz (green), and 750 Hz (purple).


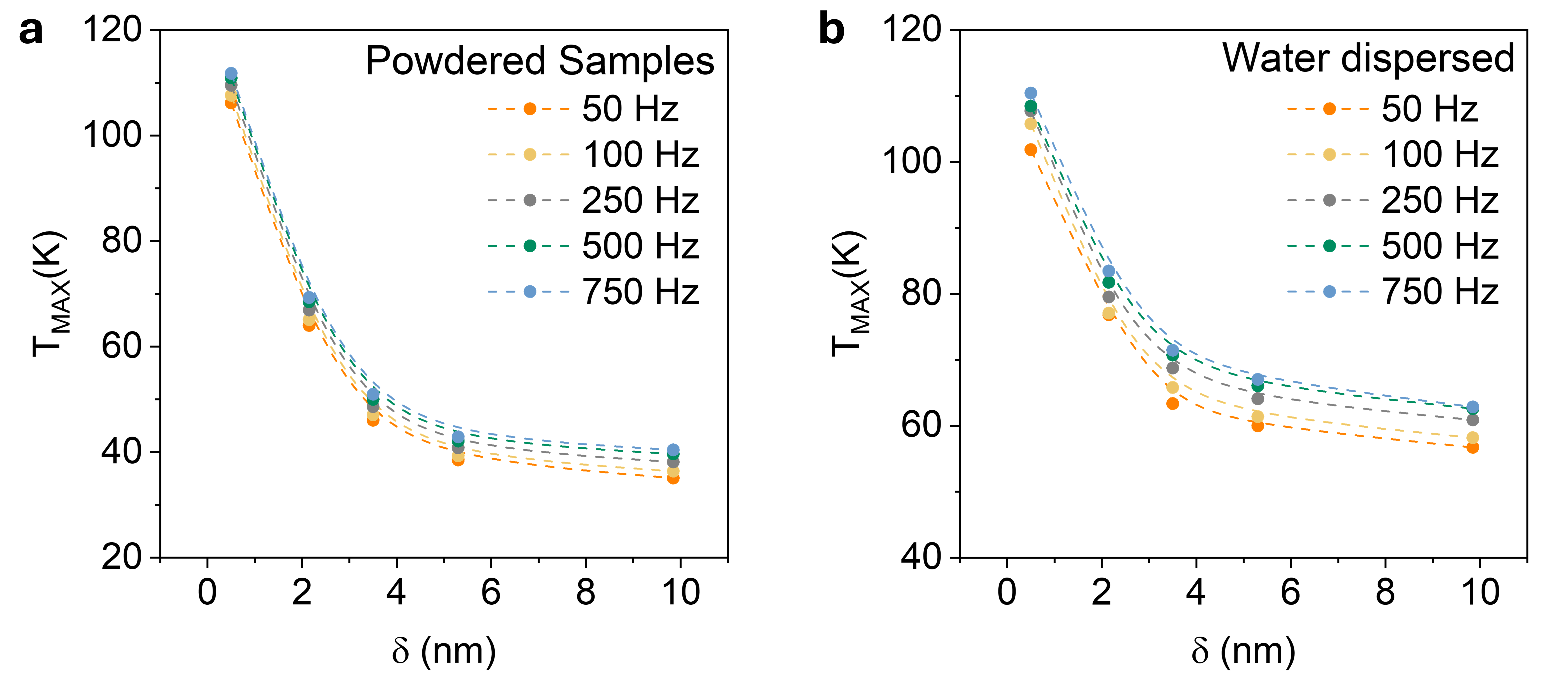


**Figure S12.** Dependence of T_MAX_, obtained from the χ’(T) curves, with spacer thickness, δ, at different frequencies: 50 Hz (orange), 100 Hz (yellow), 250 Hz (gray), 500 Hz (green) and 750 Hz (blue) for the **(a)** powdered and **(b)** water dispersed MNPs.


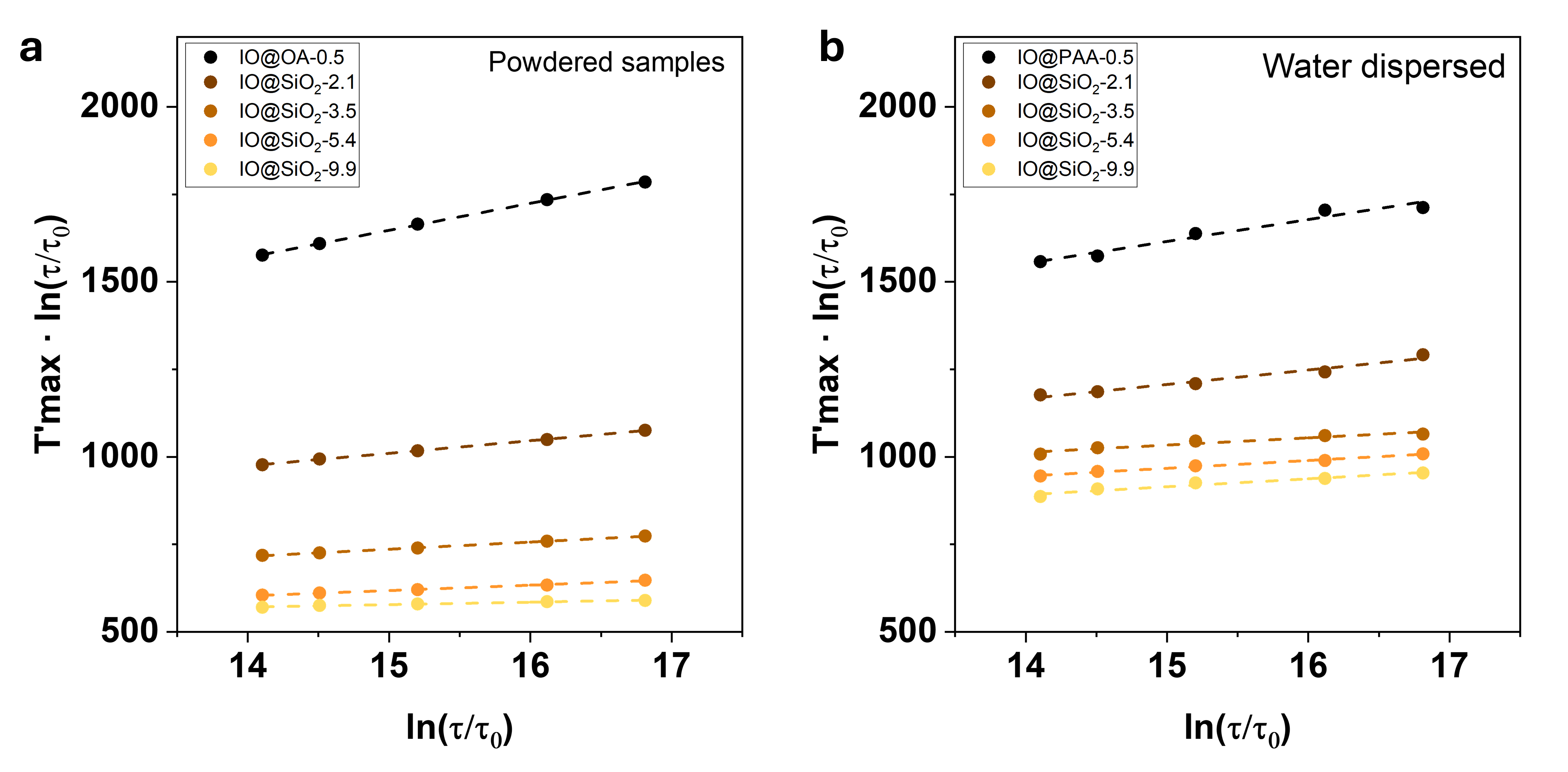


**Figure S13.** Fits to the Vogel-Fulcher equation for powder (a) and aqueous-dispersed (0.8 mM, b) MNPs with different silica shell thicknesses from δ=0.5 nm (dark tones) to δ =9.9 nm (light tones).


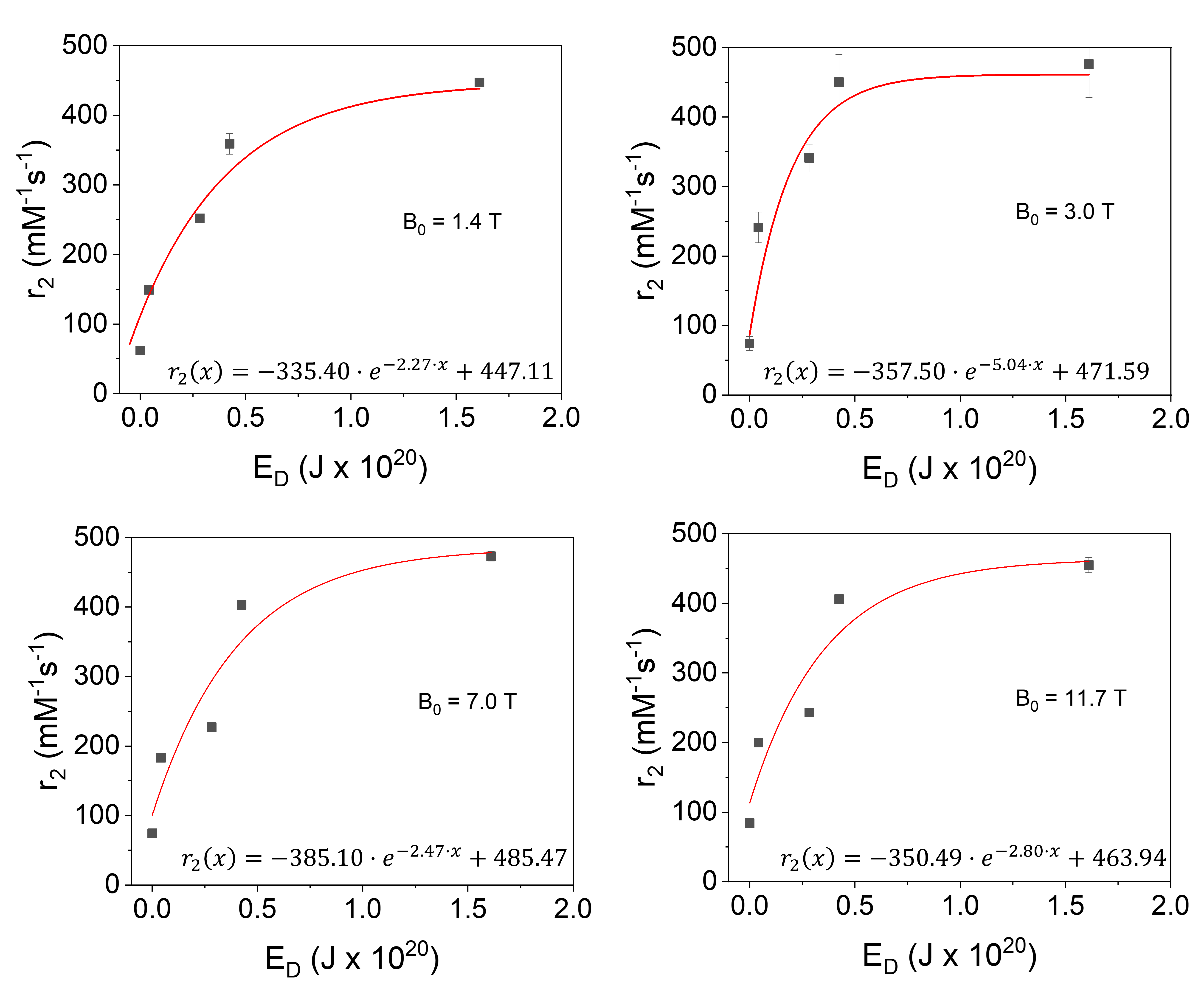


**Figure S14.** Fitting of the experimental data obtained from r_2_ with E_D_ (grey points) to the equation r_2_(x)=a⋅e^bx^+c (red line) for the different B₀ values used. From the fitting, a functional relationship was established, as indicated within the graphs.

**Quantification of dipolar interactions**

*Henkel plots*

The Henkel plots (δM) which are obtained by the ratio of the isothermal remanent magnetization (IRM, or M_R_(H)) and DC demagnetization (DCD, or M_D_(H)) were obtained through the Wohlfarth relationship:

$$M_{D} \left( H \right)= M_{\mathrm{RS}}-2M_{R}\left( H \right) (S1)$$

Where M_D_ is the DC demagnetization, M_RS_ is the saturation remanence, M_R_ is the isothermal remanence magnetization and H is the applied magnetic field. Obtaining δM from the relation:

$$M \left( H \right)=M_{D}\left( H \right)-\left[ M_{\mathrm{RS}}-2M_{R}\left( H \right) \right] (S2)$$

From these curves the Henkel plots were computed.

*E_D_ obtention from ZFC-FC protocols*

The dipolar energy term was obtained from ZFC-FC measurements, based on the Arrhenius-Néel law:

$$\tau_{N}=\tau_{0}\exp\left( \frac{\Delta E}{k_{B}T} \right) \to\Delta E= ln\left( \frac{{}_{N}}{{}_{0}} \right) k_{B}T (S3)$$

Where ΔE is given by the contribution of three energy terms: the anisotropy energy (E_A_​), the energy due to the external field (E_H_) and the E_D_. Considering that E_H_ is negligible, and accounting for ln (τ_m_/τ_0_) being approximately 25 for DC measurements [19], equation (1) can be described as follows:

$$E_{\mathrm{DC}}\approx25 k_{B}T_{B}= E_{D,i}+EA=25k_{B}T_{B,i}+EA (S4)$$

In this way, E_D_ ​ can be obtained for each value of δ from the corresponding T_B_​ value derived from ZFC-FC protocols:

$$E_{D,i}=E_{\mathrm{DC}} -EA=25k_{B}T_{B,i} -25k_{B}T_{B,min} (S5)$$

Where T_B,min_ is the value obtained for the maximum δ, corresponding to a value of minimal dipolar interactions.

*T_0_​ parameter from AC measurements (Vogel-Fulcher law)*

The Vogel-Fulcher law allows the quantification of interactions through the determination of the T_0_​ parameter, which represents the temperature at which relaxation time diverges due to interaction-driven freezing. This law describes the temperature dependence of relaxation times in interacting superparamagnetic systems, following:

$$\tau= \tau_{0}\exp\left( \frac{E_{A}}{k_{B}\left( T_{\max}-T_{0} \right)} \right) (S6)$$

where T_max_ is the temperature of the AC susceptibility peak and $\tau_{0}$ = 10^-9^ s. The T'_max_·ln (τ/τ_0_) vs ln (τ/τ_0_) plot was used to extract T_0_, which determines the intensity of dipolar interactions, ranging from low (with T_0_ close to zero) to high (as T_0_ increases).
